# The blast pathogen effector AVR-Pik binds and stabilizes rice heavy metal-associated (HMA) proteins to co-opt their function in immunity

**DOI:** 10.1371/journal.ppat.1012647

**Published:** 2024-11-18

**Authors:** Kaori Oikawa, Koki Fujisaki, Motoki Shimizu, Takumi Takeda, Keiichiro Nemoto, Hiromasa Saitoh, Akiko Hirabuchi, Yukie Hiraka, Naomi Miyaji, Aleksandra Białas, Thorsten Langner, Ronny Kellner, Tolga O Bozkurt, Stella Cesari, Thomas Kroj, Mark J Banfield, Sophien Kamoun, Ryohei Terauchi

**Affiliations:** 1 Iwate Biotechnology Research Center, Kitakami, Iwate, Japan; 2 The Sainsbury Laboratory, University of East Anglia, Norwich Research Park, Norwich, United Kingdom; 3 Department of Life Sciences, Imperial College, London, United Kingdom; 4 University of Montpellier, CIRAD, INRAE, Supagro, BGPI, Montpellier, France; 5 Department of Biochemistry and Metabolism, John Innes Centre, Norwich Research Park, Norwich, United Kingdom; 6 Laboratory of Crop Evolution, Kyoto University, Kyoto, Japan; University of Nebraska-Lincoln, UNITED STATES OF AMERICA

## Abstract

Intracellular nucleotide-binding domain and leucine-rich repeat-containing (NLR) receptors play crucial roles in immunity across multiple domains of life. In plants, a subset of NLRs contain noncanonical integrated domains that are thought to have evolved from host targets of pathogen effectors to serve as pathogen baits. However, the functions of host proteins with similarity to NLR integrated domains and the extent to which they are targeted by pathogen effectors remain largely unknown. Here, we show that the blast fungus effector AVR-Pik binds a subset of related rice proteins containing a heavy metal-associated (HMA) domain, one of the domains that has repeatedly integrated into plant NLR immune receptors. We find that AVR-Pik binding stabilizes the rice small HMA (sHMA) proteins OsHIPP19 and OsHIPP20. Knockout of *OsHIPP20* causes enhanced disease resistance towards the blast pathogen, indicating that *OsHIPP20* is a susceptibility gene (*S*-gene). We propose that AVR-Pik has evolved to bind HMA domain proteins and co-opt their function to suppress immunity. Yet this binding carries a trade-off, it triggers immunity in plants carrying NLR receptors with integrated HMA domains.

## Author summary

Rice blast disease, caused by the fungus *Magnaporthe oryzae*, is one of the most devastating diseases of rice. Therefore, understanding the mechanisms of blast fungus infection and resistance of rice against the disease is important for global food security. In this study, we show that the *M*. *oryzae* effector protein AVR-PikD binds rice sHMA proteins and stabilizes them, presumably to enhance pathogen infection. We show that loss-of-function mutants in one rice sHMA, OsHIPP20, reduced the level of susceptibility against a compatible isolate of *M*. *oryzae*, suggesting that *M*. *oryzae* requires host sHMA to facilitate invasion. Remarkably, *OsHIPP20* knockout rice line showed no growth defect, suggesting editing sHMA genes may present a novel source of resistance against blast disease.

## Introduction

Plant pathogens target host processes to promote disease by secreting effector proteins [1]. Some of the host targets of effectors have been co-opted by plant intracellular nucleotide-binding leucine rich repeat (NLR) immune receptors to act as baits to detect pathogens, and in this context are known as integrated domains (IDs) [2–5]. Genome-wide bioinformatics searches have found such domains in diverse NLR immune receptors from multiple plant families [4–7]. We hypothesize that such widespread NLR-IDs modulate basic immune responses that are conserved among plants. Examples of NLR-IDs with known functions include the WRKY-ID of Arabidopsis RRS1 [8,9] and the heavy metal-associated (HMA) domain found in four botanical families [4]. In rice, HMA domains have been integrated into two different NLRs, RGA5 and Pik-1 [10,11]. In addition to rice (a member of Poaceae), HMA domains have also been integrated into NLR immune receptors of plant species in the Brassicaceae, Fabaceae and Rosaceae [4,5]. This indicates that HMA-containing proteins have likely been repeatedly targeted by pathogens across the diversity of flowering plants. Therefore, understanding the endogenous function of HMA-containing proteins has the potential to reveal important basic features of plant disease susceptibility and immunity. In this study, we report rice HMA-containing proteins that are targets of a blast pathogen effector from *Magnaporthe* (syn. *Pyricularia*) *oryzae* and address their potential function.

The rice *Pik* locus comprises two NLR genes, *Pik-1* and *Pik-2*, and recognizes the *M*. *oryzae* effector AVR-Pik [11], triggering an immune response that restricts infection. AVR-Pik is a 113-amino-acid protein originally defined as having no sequence similarity to known protein domains [12]. More recently, structure-informed similarity searches showed that AVR-Pik belongs to the MAX (*Magnaporthe* AVRs and ToxB-like) family of fungal effectors [13], which adopt a six β-sandwich fold stabilized by buried hydrophobic residues, and commonly but not always, a disulfide bond. Pik-1 recognition of AVR-Pik is mediated by direct binding of the effector to an HMA domain [14] located between the N-terminal coiled-coil (CC) and nucleotide binding (NB) domains of Pik-1 [15,16]. *AVR-Pik* and *Pik-1* are described as being involved in a coevolutionary arms race that has resulted in the emergence of allelic series of both effector genes in the pathogen and NLR genes in the host [15,17,18].

Biochemical and structural analysis of complexes between AVR-Pik variants and HMA domains of different Pik-1 alleles revealed the molecular interactions between the effector and NLR-ID [14,17,19]. This knowledge recently allowed structure-guided protein engineering to expand the recognition profile of a Pik NLR to different AVR-Pik variants [19,20]. The Pik-1 HMA domains exhibit a four β-sheets and two α-strands (βαββαβ) topology similar to the yeast copper transporter domain Ccc2A [21], even though the characteristic MxCxxC metal-binding motif is degenerate in Pik-1. The integrated HMA domain of RGA5 also adopts the classical HMA domain fold but, intriguingly, uses a different interface to interact with the *M*. *oryzae* effectors AVR-Pia and AVR1-CO39 [22].

HMA domains are also found in other plant proteins that are unrelated to NLRs [23]. These proteins form large and complex families known as heavy metal-associated plant proteins (HPPs) and heavy metal-associated isoprenylated plant proteins (HIPPs), here collectively referred to as small proteins containing an HMA domain (abbreviated as sHMA proteins). One such sHMA protein is the product of the rice blast partial resistance gene *pi21* [24]. The recessive allele *pi21*, a presumed loss-of-function allele with a deletion mutation, confers partial broad-spectrum resistance to rice against compatible isolates of *M*. *oryzae*. This finding implicates HMA domain-containing proteins in rice defense [24]. However, the molecular function of Pi21 and other rice sHMA proteins have not been characterized to date.

Unlike other *M*. *oryzae* effectors, *AVR-Pik* does not show extensive presence/absence polymorphisms within the rice-infecting lineage, and its evolution in natural pathogen populations is mainly driven by nonsynonymous amino acid substitutions [15,25]. This suggests that *AVR-Pik* encodes an activity of benefit to the pathogen that is maintained in resistance-evading forms of the effector. To address the virulence function of AVR-Pik, we set out to identify rice proteins other than the Pik NLRs that interact with this effector. We found that AVR-Pik binds and stabilizes a subset of sHMA proteins. Knockout of one sHMA gene (*OsHIPP20*) conferred enhanced resistance to infection by the blast pathogen, suggesting *OsHIPP20* is a susceptibility gene (S-gene). Our model is that AVR-Pik effectors interfere with sHMA function by stabilizing and relocating these proteins to support pathogen invasion.

## Results

### AVR-PikD binds members of a subclade of small heavy metal associated proteins (sHMAs) of rice

To identify rice proteins that may be putative targets of AVR-PikD, we performed a yeast 2-hybrid screen (Y2H) with the effector as bait and a cDNA library prepared from leaves of rice cultivar Sasanishiki inoculated with *M*. *oryzae* as the prey. From this screen we identified four HMA-containing proteins, named OsHIPP19 (LOC_Os04g39350), OsHIPP20 (LOC_Os04g39010), OsHPP04 (LOC_Os02g37300) and OsHPP03 (LOC_Os02g37290) [23], as interactors of AVR-PikD, amongst other proteins (**S1 Table**). The sizes of AVR-PikD interacting HMAs ranged from 118 (OsHPP03) to 123 (OsHIPP19) amino acids.

**Fig 1:**
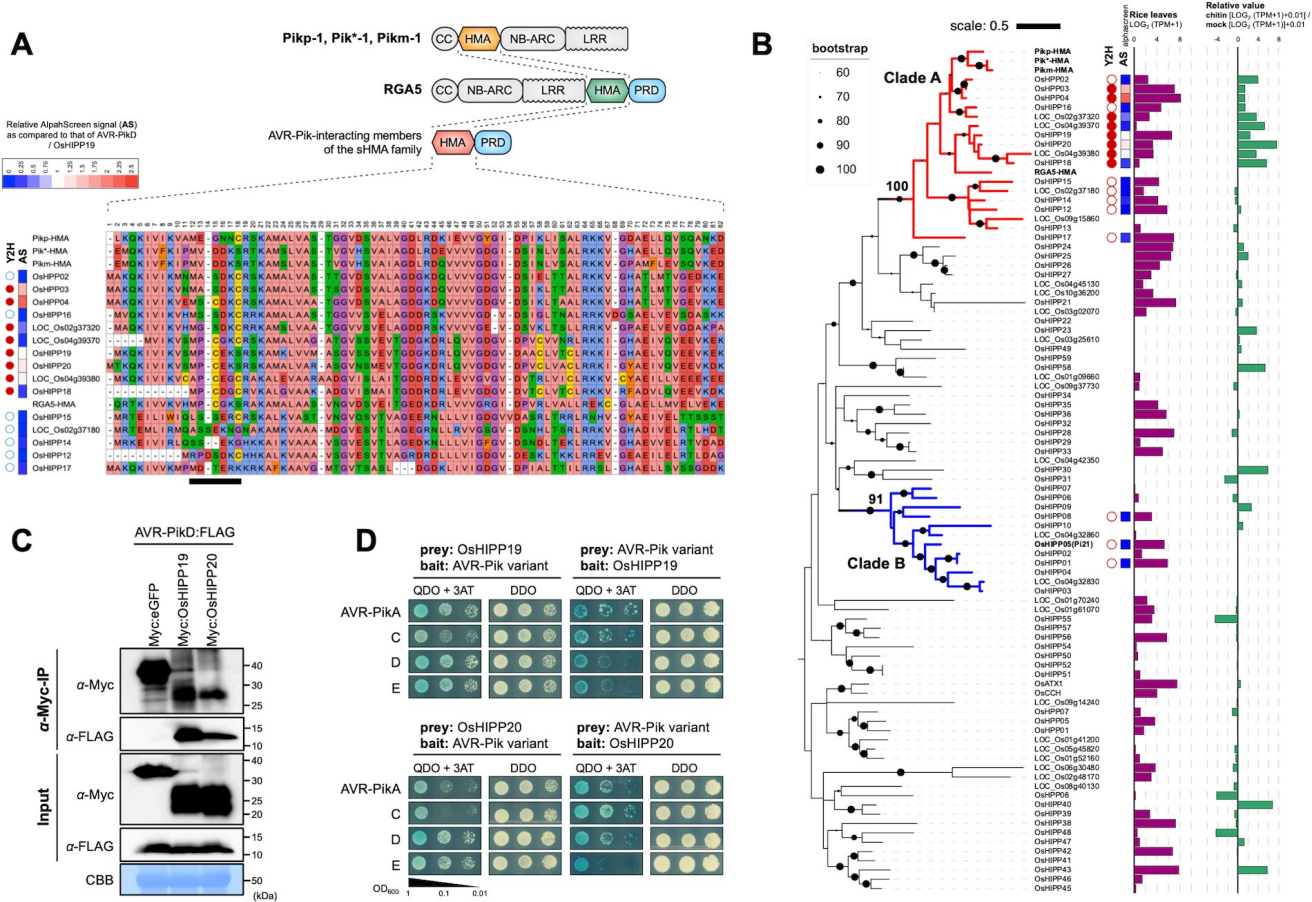
AVR-PikD binds Clade A sHMAs. **(A)** Schematic representation of the Pik-1 (Pikp-1, Pik*-1, Pikm-1) and RGA5 Nucleotide-binding Leucine Rich Repeat Receptors (NLRs) and small HMA (sHMA) proteins of rice. CC: coiled-coil domain; NB-ARC: nucleotide binding domain; LRR: leucine rich repeat; PRD: proline-rich domain. Amino acid sequence alignment of a subset of HMA proteins of rice. The black bar highlights the putative metal-binding motif MxCxxC. Interaction with AVR-PikD is indicated for yeast two-hybrid (Y2H: red dot: binding; white dot: non-binding) and AlphaScreen (AlphaScreen Signal [AS]: strength of interaction signal as compared to that of AVR-PikD/OsHIPP19 interaction is given in the inset). **(B)** A maximum likelihood tree of the HMA domains of 87 sHMA of rice. Amino acid sequences of the HMA domains were aligned and used for reconstruction of the phylogenetic tree. The dots on the branches indicate bootstrap values after 1,000 replications. Clade A and Clade B are indicated by red and blue branches, respectively. Note Pi21 (OsHIPP05) belongs to Clade B. Y2H and AlphaScreen results (AS) are as shown as in **(A)**. Bar graphs in purple color show expression level of each gene in leaves as revealed by RNA-seq (log2(TPM+1) value). Bar graphs in green color show the induction level of each gene in rice suspension cultured cells after chitin treatment (log2[TPM of chitin-treated cultured cells+1] + 0.01)/ (log2[TPM of mock-treated cultured cells+1] + 0.01). **(C)** Results of co-immunoprecipitation of AVR-PikD with OsHIPP19 and OsHIPP20 transiently expressed in *Nicotiana benthamiana* leaves. **(D)** Y2H interactions of the AVR-Pik variants, AVR-PikA, AVR-PikC, AVR-PikD, AVR-PikE, to OsHIPP19 and OsHIPP20.

Rice sHMA proteins typically comprise a conserved N-terminal HMA domain followed by a variable proline-rich domain (**Fig 1A**) and may contain a C-terminal “CaaX” isoprenylation motif (where “a” represents an aliphatic amino acid and X represents any amino acid). They form a large protein family with 87 members in the rice genome (cultivar Nipponbare) as annotated by Rice Genome Annotation Project [26] (**Fig 1B**). Phylogenetic analyses of the aligned HMA domains of rice sHMA proteins revealed two clades supported by high bootstrap values (> 90%) that we designate here as Clades A and B. All four sHMA proteins interacting with AVR-PikD belong to Clade A (**Fig 1B**). Interestingly, the HMA domains of RGA5 and three alleles of the Pik-1 NLRs also cluster in Clade A. However, the integrated HMA domains of Pik-1 (Pik*-HMA, Pikm-HMA and Pikp-HMA) and RGA5 (RGA5-HMA) are on separate branches in the tree, indicating distinct lineages and diversification patterns.

To determine if AVR-PikD interacts with other Clade A sHMA proteins, we selected 15 that are expressed in rice leaves (**Fig 1B**) and tested pairwise interactions by Y2H (**S1 Fig**). This experiment showed that AVR-PikD binds around half of the tested Clade A sHMA proteins (**Fig 1B**). We also tested binding of AVR-PikD with three sHMA proteins from Clade B, including OsHIPP05 (Pi21). These sHMA proteins did not bind AVR-PikD (**S1 Fig**), revealing that AVR-PikD shows specific binding to Clade A sHMA proteins (**Fig 1B**). These results were overall confirmed using the AlphaScreen (Amplified Luminescent Proximity Homogenous Assay Screen) method [27,28] (**Figs 1B and S2**). Interestingly, RNA-seq analysis indicated that AVR-PikD-interacting sHMAs were induced by chitin treatment of rice suspension cultured cells (**Fig 1B**), suggesting potential roles in immunity.

We also tested the interaction of Clade A sHMA proteins OsHIPP19, OsHIPP20, OsHPP04, OsHPP03 and LOC_Os04g39380 with the *M*. *oryzae* effectors AVR-Pia and AVR1-CO39, which interact with the HMA domain of RGA5 [16]. We found that AVR-Pia and AVR1-CO39 did not bind any of the sHMAs tested (**S3 Fig**). Also, none of the three AVRs interacted with the Pi21 HMA protein (**S3 Fig**). Interactions between AVR-PikD and OsHIPP19 and OsHIPP20, the top two most frequently recovered sHMA proteins in the initial Y2H assay (**S1 Table**), were further confirmed by co-immunoprecipitation using proteins expressed in *N*. *benthamiana* (**Fig 1C**).

Naturally occurring AVR-Pik variants are differentially recognized by allelic Pik NLRs. These recognition specificities correlate with the binding affinity of AVR-Pik variants to the integrated HMA domain of the Pik-1 NLR [14,15,17,19]. We tested whether the AVR-Pik variants AVR-PikA, C, or E, interact with the rice sHMA proteins OsHIPP19 and OsHIPP20 in Y2H. The results of this experiment (**Figs 1D and S4**) showed that similar to AVR-PikD, all AVR-Pik variants tested interacted with OsHIPP19 and OsHIPP20. This result suggests that all the tested AVR-Pik variants bind sHMAs, the possible host target proteins, whereas they vary in the recognition by different alleles of Pik NLRs. After posting the first version of this manuscript to bioRxiv in 2020, Maidment et. applied gel filtration assay and *Surface Plasmon Resonance* (*SPR) assay* and confirmed that OsHIPP19 binds AVR-PikD, C and F with nanomolar affinity [29].

### Three regions of sHMAs have close contact with AVR-PikD and model-guided structure prediction allowed converting AVR-Pik non-binding sHMAs to AVR-Pik-binding

**Fig 2:**
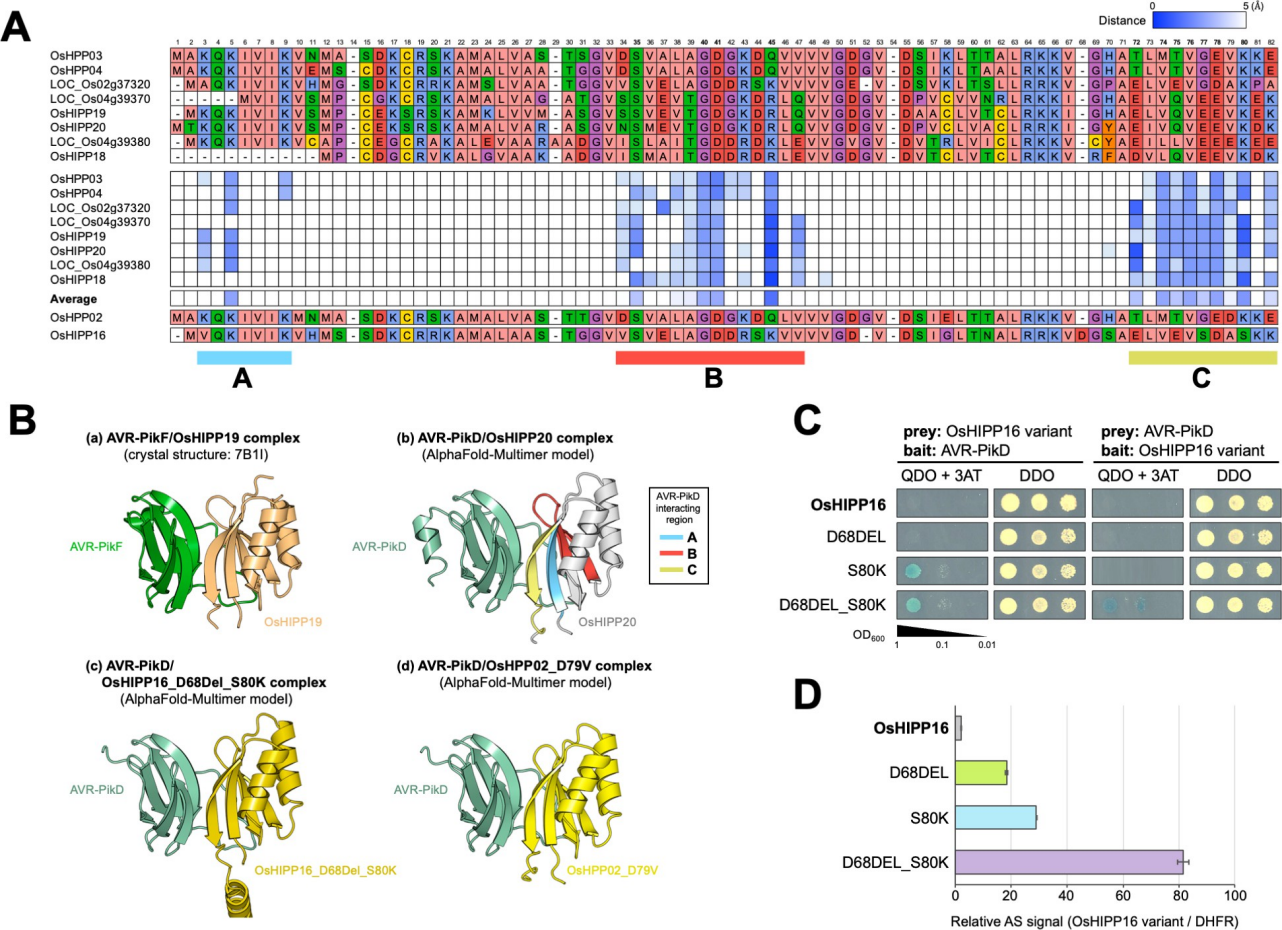
Three regions of sHMAs are predicted to have close contact with AVR-PikD. **(A)** Amino acid sequence alignment of sHMAs (top) and a matrix of distance of each amino acid residue to AVR-PikD protein as predicted by ColabFold (bottom). Predicted atomic distance below 5Å are indicated by blue tiles. Three regions indicated by A, B and C are predicted to be in close contact with AVR-PikD. **(B)** Binding structures between AVR-PikF (green) and OsHIPP19 (light orange) (a: Crystal structure; Maidment et al. 2021) [29], predicted structure between AVR-PikD (light green) and OsHIPP20 (grey) (b: AlphaFold-multimer model), predicted structure between AVR-PikD (light green) and OsHIPP16_D68DELS80K (dark yellow) (c: AlphaFold-multimer model), and predicted structure between AVR-PikD (light green) and OsHPP02_D79V (yellow) (d: AlphaFold-multimer model). **(C)** Y2H interactions between the variants of OsHIPP16 (OsHIPP16, OsHIPP16_D68DEL, OsHIPP16_S80K, OsHIPP16_D68DEL_S80K) and AVR-PikD. **(D)** AlphaScreen interactions between the variants of OsHIPP16 (OsHIPP16, OsHIPP16_D68DEL, OsHIPP16_S80K, OsHIPP16_D68DEL_S80K) and AVR-PikD. The values are relative AlphaScreen signals (AS) to that of OsHIPP16/DHFR interaction signal (negative control). The error bars represent SD of 3 replications.

A previous study by Maidment et al. determined the crystal structure of the AVR-PikF/OsHIPP19 complex [29], which was similar to that of AVR-PikD/Pikp1-HMA [14] and described three predominant interfaces. We took advantage of the multiple interactions we identified in our Y2H screen to further investigate AVR-PikD/sHMA complexes using computational structural biology. We predicted AVR-PikD/sHMA complex models using ColabFold v1.5.2 (AlphaFold2 using MMseqs2) [30], which revealed that three regions A, B, C conserved in Clade A sHMA seem closely (Atomic distance < 5Å) positioned to AVR-PikD (**Figs 2A and S5**). These regions correspond to the interfaces 1, 2, 3, respectively, as reported by De la Concepcion et al. [17] and Maidment et al. [29]. To validate the importance of these interfaces, we selected OsHIPP16, an sHMA in Clade A that does not bind AVR-PikD in Y2H (**S1 Fig**) or AlphaScreen (**S2 Fig**), for further investigation. Modeling of the interaction predicted that OsHIPP16 does not form a complex with AVR-PikD in the way experimentally determined for OsHIPP19/AVR-PikF or predicted for OsHIPP20/AVR-PikD (**Figs 2A and S5C**). We compared the amino acid sequences of OsHIPP16 and OsHIPP20 and found that the former has an insertion of an aspartic acid (D) at position 68, and has a serine (S) at position 80 where a lysine (K) is present in OsHIPP20 (**Fig 2A**). Modeling indicated a deletion of D68 (resulting in OsHIPP16-D68DEL) and an amino acid replacement S80K (OsHIPP16-S80K) could render OsHIPP16 capable of binding AVR-PikD (**Figs 2B and S6**). In a Y2H assay, OsHIPP16-D68DEL did not bind AVR-PikD, but OsHIPP16-S80K bound the effector, and OsHIPP16-D68DEL/S80K strongly bound (**Figs 2C and S7**). In an AlphaScreen experiment, each single mutant weakly bound AVR-PikD, but OsHIPP16-D68DEL/S80K strongly bound AVR-PikD (**Fig 2D).** We also tested a second AVR-PikD non-binding sHMA, OsHPP02 (**S1 and S2 Figs**) to see whether we could obtain a gain-of-binding mutant for this protein. Modeling of the interaction predicted that OsHPP02 does not form an OsHIPP20/AVR-PikD-like complex (**S5C Fig**), but may be converted to AVR-PikD-binding by a single amino acid change at position 79 (within region C) from an aspartic acid (D) to the valine (V) found in OsHIPP20 (**Figs 2A and S8**). Indeed, OsHPP02-D79V bound to AVR-PikD in both Y2H and AlphaScreen assays (**S8 Fig**). Overall, these results, obtained by model-based interaction prediction, confirm the findings previously observed using the AVR-PikF/OsHIPP19 crystal structure [29] that region C conserved in Clade A sHMAs is important for the interactions between AVR-PikD and Clade A sHMAs.

### AVR-PikD stabilizes OsHIPP19 and OsHIPP20 and affects their subcellular localization

**Fig 3:**
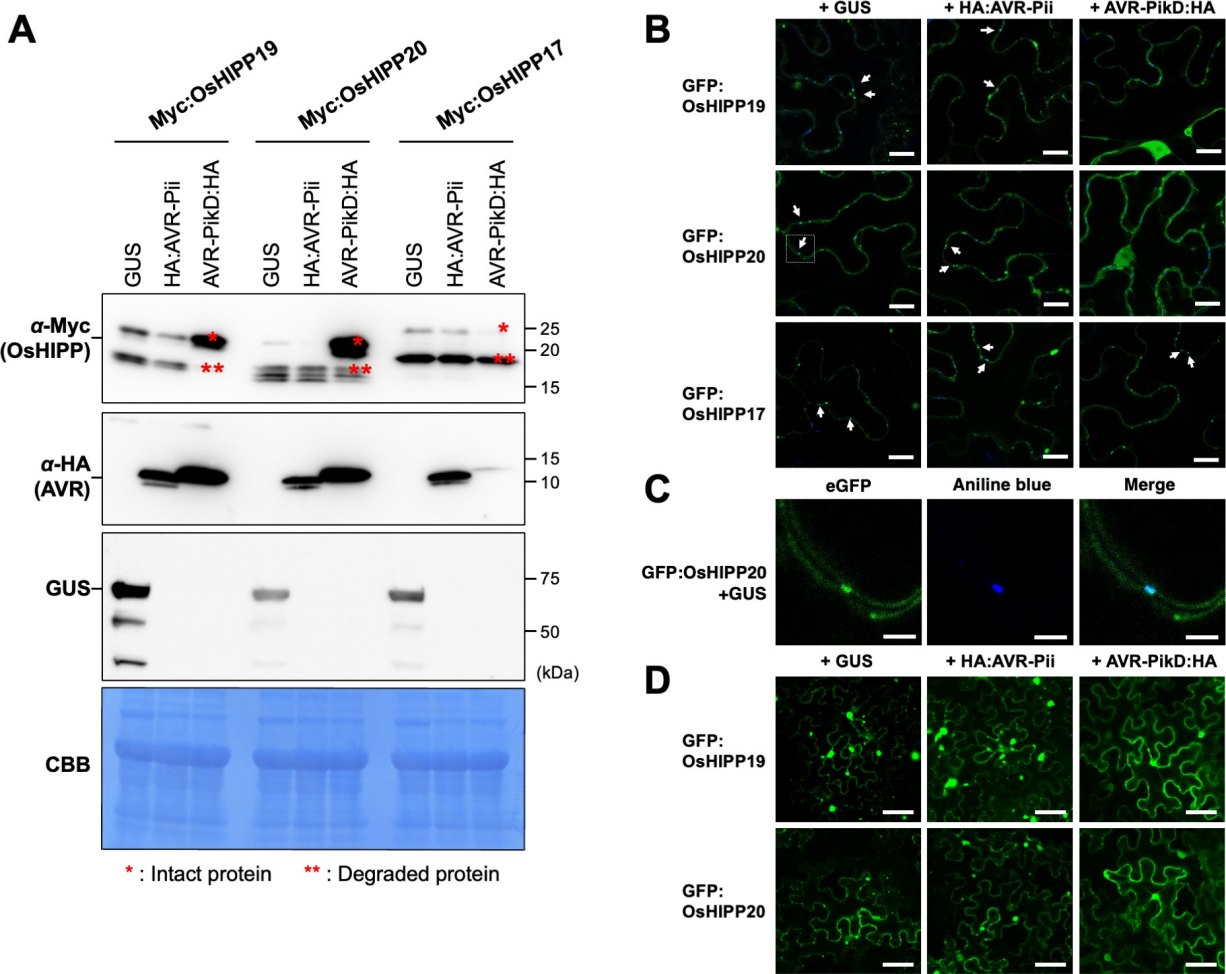
AVR-PikD stabilizes sHMA proteins and alters sHMA subcellular localization in *N*. *benthamiana*. **(A)** sHMA proteins (Myc:OsHIPP19, Myc:OsHIPP20 and Myc:OsHIPP17) were transiently expressed in *N*. *benthamiana* leaves together with either GUS, HA:AVR-Pii or AVR-PikD:HA and were detected by an anti-Myc antibody. The result for supernatant fraction after fractionation of leaf extract is shown. The result for pellet fraction is shown in **S9 Fig**. OsHIPP19 and OsHIPP20 bound by AVR-PikD remain largely stable, whereas OsHIPP19 and OsHIPP20 expressed with GUS or AVR-Pii were degraded to a lower mass fragment. OsHIPP17 binds AVR-PikD only weakly and is degraded even in the presence of the effector. AVR-PikD seems unstable when unbound to target proteins. **(B)** GFP:OsHIPP19 and GFP:OsHIPP20 seem to accumulate at plasmodesmata. Plasmodesmata are stained by aniline blue (blue color). White arrows indicate colocalization of GFP and aniline blue (Cyan color). Co-expression of AVR-PikD:HA relocates GFP:OsHIPP19 and GFP:OsHIPP20 from plasmodesmata. GFP:OsHIPP17 co-expressed with AVR-PikD:HA shows no relocation. Scale bar: 20 μm. **(C)** A magnified view of GFP-OsHIPP20 in an inset square of **(B)** for GFP (left), aniline blue (center) and merged view of GFP and aniline blue (right). Scale bar: 5 μm. **(D)** GFP:OsHIPP19 and GFP:OsHIPP20 accumulate to punctae-like structures in the cells when expressed with GUS or HA:AVR-Pii, whereas these proteins were evenly distributed in the cytoplasm when expressed with AVR-PikD:HA. We obtained similar results in three independent experiments. Scale bar: 200 μm.

Next, we aimed to determine the effect of AVR-PikD binding on the putative function sHMA proteins. Firstly, we co-expressed the N-terminally Myc-tagged OsHIPP19 and OsHIPP20 with AVR-PikD in *N*. *benthamiana* leaves by agroinfiltration (see Materials and Methods). Following expression, leaf extract was separated into supernatant and pellet fractions by centrifugation, and each fraction was analyzed by western blot (**Figs 3A and S9**). We used expression of β-glucuronidase (GUS) protein and AVR-Pii, a *M*. *oryzae* effector unrelated to AVR-Pik, as controls that do not bind OsHIPP19 and OsHIPP20. In the supernatant fraction we observed that the OsHIPP19 and OsHIPP20 proteins were degraded to smaller fragments when co-expressed with GUS or AVR-Pii. However, when co-expressed with AVR-PikD, we detected stronger signals of intact OsHIPP19 and OsHIPP20. As an additional control we also tested OsHIPP17, an sHMA that does not bind AVR-PikD in either Y2H or by in planta coimmunoprecipitation, and only weakly interacts with AVR-PikD by the AlphaScreen method (**Figs 1, 3A, S2 and S10)**. We found that OsHIPP17 was degraded to a smaller fragment even in the presence of AVR-PikD. These results show that the AVR-PikD effector stabilizes sHMA proteins OsHIPP19 and OsHIPP20 in the plant cytosol, and this stabilization is specific to sHMA proteins that interact with the effector. We also noted consistently observed lower accumulation of AVR-PikD in the supernatant when co-expressed with OsHIPP17. Interestingly, OsHIPP19, OsHIPP20 and OsHIPP17 proteins in the pellet fraction were not degraded to lower molecular fragments irrespective of the presence or absence of AVR-Pik (**S9 Fig**), suggesting that the membrane-anchored versions of the sHMAs are maintained as intact proteins.

Next, we tested whether binding of AVR-PikD affected the subcellular localization of specific sHMA proteins. We transiently co-expressed the N-terminally GFP-tagged OsHIPP19, OsHIPP20 and OsHIPP17 (GFP:OsHIPP19, GFP:OsHIPP20 and GFP:OsHIPP17) by agroinfiltration in *N*. *benthamiana* leaves together with either GUS, AVR-Pii or AVR-PikD, and performed confocal microscopy as described in Methods. Interestingly, GFP:OsHIPP20 localized to dot-like structures in the cell wall. To further investigate these membrane puncta, we tested for co-localization with aniline blue, a known marker for callose deposition commonly associated with plasmodesmata [31] (**Fig 3B**). Interestingly, when we co-expressed AVR-PikD with the HIPPs, GFP:OsHIPP19 and GFP:OsHIPP20 were relocalized from plasmodesmata to the cytosol (**Figs 3B and S11**). Also, when the cells were observed in a lower magnification, we found that GFP:OsHIPP19 and GFP:OsHIPP20 showed nucleo-cytoplasmic localization and accumulated in punctate structures with varying sizes in the presence of GUS or AVR-Pii (**Figs 3C and S12**). When OsHIPP19 or OsHIPP20 was co-expressed with AVR-PikD, these punctae-like structures were not observed and the sHMA proteins were diffused in the cytoplasm and nucleus. Our western-blot analysis showed that OsHIPP19 and OsHIPP20 in the pellet fraction are intact (**S9 Fig**), suggesting that GFP signal in the dot-like structure and punctae represent the intact proteins anchored to the membranes. We hypothesize that AVR-PikD binding alters the stability and the subcellular distribution of sHMA proteins.

### *OsHIPP20* is a susceptibility gene to *Magnaporthe oryzae*

**Fig 4:**
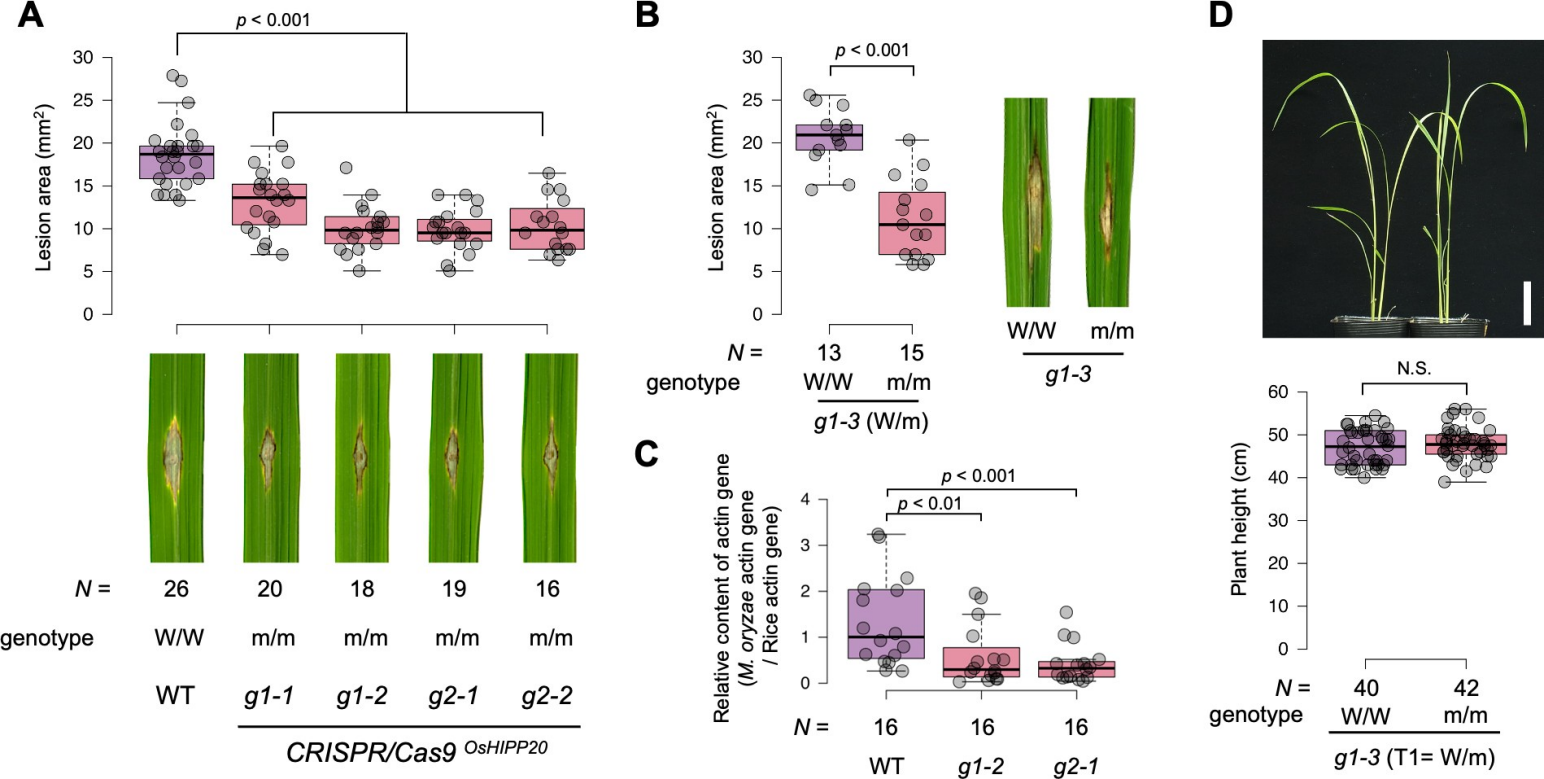
*OsHIPP20* is a susceptibility gene (S-gene). **(A)** Results of punch inoculation of conidia of a compatible isolate Sasa2 to the wild-type Sasanishiki (WT) and T2 generation of two homozygous KO rice groups (guide RNA-1 [g1] and guide RNA-2 [g2], each with two replicates [g1-1, g1-2 and g2-1 and g2-2]). Box plots show lesion area sizes in the rice lines (top). Statistical significance is shown after Wilcoxon rank sum test. Photos of typical lesions developed on the leaves after inoculation of *M*. *oryzae* (bottom). The number (***N***) of leaves used for experiments are indicated below. **(B)** Results of punch inoculation of Sasa2 conidia to T2 progeny segregated to the wild type allele (W/W) and KO-type allele (m/m) from a T1 heterozygous KO line (g1-3). Statistical significance is shown according to Wilcoxon rank sum test. **(C)** Results of spray inoculation of conidia of a compatible isolate Ken53-33 to the wild-type Sasanishiki (WT) and T2 generation of the *OsHIPP20*-knockout lines g1-2 and g2-1. Box plots show the relative content of fungal actin gene DNA (*M*. *oryzae* actin gene DNA / rice actin gene DNA) as determined by quantitative PCR. Statistical significance is shown according to Wilcoxon rank sum test. **(D)** No growth defect in *OsHIPP20* KO line as compared to the wild type one month after seed sowing. Top: Overview of the W/W and m/m plants. Bottom: Box plot showing plant height distribution of W/W and m/m plants segregated from the g1-3 heterozygous KO line. Statistical significance is shown according to Wilcoxon rank sum test. Scale bar: 5cm.

Of the seven sHMA proteins that interacted with AVR-PikD by Y2H, we selected OsHIPP19, OsHIPP20 and OsHPP04 for further study as were most frequently identified in this screen (**S1 Table**), and showed strong interaction profiles with AVR-PikD (**Fig 1**). To explore the function of these sHMA proteins in rice, we generated knockout (KO) mutants by CRISPR/Cas9-mediated mutagenesis in the rice cultivar Sasanishiki, which is susceptible to the blast fungus isolates Sasa2 and Ken53-33. We targeted *OsHIPP19*, *OsHIPP20* and *OsHPP04* (**Figs 4A and S13**). The resulting KO lines were challenged with the *M*. *oryzae* isolate Sasa2. The KO lines of *OsHIPP19* (two independent lines) and *OsHPP04* showed a similar level of infection as the wild-type control (Sasanishiki) after punch inoculation of the conidia of Sasa2 (**S13 Fig**). However, the *OsHIPP20* KO line showed a reduction in lesion size caused by *M*. *oryzae* infection after the punch inoculation of Sasa2 conidia (**Fig 4A**). In addition, a Sasanishiki line heterozygous for *OsHIPP20* wild-type and KO alleles were self-fertilized and its progeny were challenged with *M*. *oryzae* Sasa2. Progeny with homozygous *OsHIPP20* KO allele showed enhanced resistance as compared to their sib lines with *OsHIPP20* wild-type alleles (**Fig 4B**). We also observed an enhanced resistance in *OsHIPP20* KO plants as compared to the wild type in a spray inoculation assay of conidia of a compatible isolate Ken53-33 (**Figs 4C** and S14). We confirmed there was no off-target editing in other sHMA genes close to *OsHIPP20* by whole genome resequencing (**S2 Table**). These results indicate that *OsHIPP20* is a susceptibility (*S-*) gene that is required for full infection of rice (cultivar Sasanishiki) by *M*. *oryzae*. Growth of *OsHIPP20*-KO Sasanishiki lines was comparable to the wild-type Sasanishiki (**Figs 4D and S15B**). To evaluate the utility of *OsHIPP20*-KO line for practical use, a thorough assessment of various traits including yield is still required.

## Discussion

In this paper, we set out to identify host targets of the *M*. *oryzae* effector AVR-Pik, to explore the potential virulence function of this effector. We found that AVR-Pik binds multiple sHMA proteins of rice that belong to the same phylogenetic clade (Clade A), which also contain the integrated HMA domains of Pik-1 and RGA5 (**Fig 1**). These findings support the view that NLR integrated domains have evolved from the host targets of pathogen effectors and that the HMA-containing proteins induced by chitin, a pathogen Pathogen-Associated Molecular Pattern (PAMP), are a major host target of plant pathogen effectors. In an independent study, Maidment et al. showed that AVR-Pik binds to OsHIPP19 with nanomolar affinity *in vitro* and showed the interaction of the effector with this sHMA is via an interface conserved with the Pik-1 integrated HMA domains providing further evidence that this effector targets host sHMA proteins [29].

Heavy metal-associated (HMA) domains were first defined in metal binding domains of P-type ATPase family copper transport proteins, including human MNK and WND proteins, mutations of which cause Menkes disease and Wilson disease, respectively [32]. HMA domains are also found in a number of heavy metal transport or detoxification proteins both in bacteria and eukaryotes. The yeast metallochaperone Atx1 was shown to deliver monovalent copper ions to the P-type ATPase Ccc2 that transports copper to trans-Golgi vesicle where it is taken up by the multicopper oxidase Fet3 [33–35]. A typical HMA domain contains two conserved cysteine residues involved in metal binding in a MxCxxC motif that is located towards the N-termini of the domain [32].

In most organisms, only a small number of HMA-containing proteins have been reported. By contrast, in plants, proteins containing HMA-like domains have massively expanded [23,36,37]. For example, Barth et al. identified 44 Arabidopsis genes that encode for proteins containing an HMA domain and a C-terminal putative isoprenylation motif (CaaX) [37]. Based on the presence or absence of the C-terminal isoprenylation motif, De Abreu-Neto et al. grouped plant sHMAs into heavy metal-associated isoprenylated plant proteins (HIPPs) and heavy metal-associated plant proteins (HPPs) [23]. We have chosen to use the naming convention of De Abreu-Neto et al. here [23]. In this manuscript, we present an analysis of the HMA-like repertoire of the rice (cultivar Nipponbare) genome, revealing the presence of at least 87 HMA-containing small protein (abbreviated as sHMA) genes (**Fig 1**).

The biological functions of plant sHMA proteins reported so far are diverse. Two Arabidopsis HMA-containing proteins, CCH and ATX1, complemented yeast *atx1* mutant, and are presumed to be involved in copper transport [38–40]. Barth et al. showed that the Arabidopsis HMA-containing protein HIPP26 localizes to nuclei and interacts with a zinc-finger transcription factor ATHB29 [37], while Gao et al. reported the same protein (with an alternative name, ATFP6) was localized to plasma membrane and interacted with acyl-CoA–binding protein ACBP2 [41], which was hypothesized to be involved in membrane repair after oxidative stress. Zhu et al. reported that the Arabidopsis HMA-containing protein NaKR1 interacts with Flowering Locus T (FT) and mediates its translocation from leaves to shoot apices [42]. Cowan et al. reported that potato mop-top virus (PMTV) movement protein TGB1 interacts with *Nicotiana benthamiana* sHMA protein HIPP26 and relocalizes this protein from the plasma membrane to the nucleus, thus contributing to PMTV long-distance movement by altering transcriptional regulation [43].

Genetic studies have also revealed roles of specific plant sHMA proteins in defense and susceptibility towards pathogens. Deletion in the proline-rich domain of Pi21, a rice sHMA, conferred a partial resistance against compatible isolates of *M*. *oryzae* [24]. Virus-induced gene silencing of wheat *TaHIPP1* enhanced resistance against stripe rust caused by *Puccinia striiformis* f. sp. *Triticii* [44]. Similarly, a knockout mutant of *Arabidopsis AtHMAD1* enhanced resistance against virulent *Pseudomonas syringae* DC3000 [45] and a knockout mutant of *Arabidopsis AtHIPP27* enhanced resistance against beet cyst nematode [46]. However, it remains unclear how these sHMA proteins impact interactions with these diverse pathogens. Nonetheless, given that HMA domains have integrated into NLR immune receptors in at least four botanical families, it is likely that HMA containing proteins have repeatedly been targeted by pathogens across a diversity of flowering plant species and are thus important components in plant-pathogen interactions.

In addition to Pik-1, the NLR RGA5 also carries an integrated HMA domain that binds two *M*. *oryzae* effectors, AVR-Pia and AVR1-CO39. However, in our Y2H assays (with high stringency conditions), we didn’t detect any interaction between AVR-Pia and AVR1-CO39 and the tested sHMA proteins. We hypothesize that these two effectors may weakly bind the tested sHMAs or bind other rice sHMA proteins among the >80 members of this family.

In this study, we revealed that gene knockout of *OsHIPP20* confers enhanced resistance to rice against a compatible isolate of *M*. *oryzae* (**Fig 4**). Therefore, like *Pi21*, *OsHIPP20* is a susceptibility gene (*S*-gene), whose activity is required for full virulence of the *M*. *oryzae* pathogen in rice. Rice *pi21* is established as a useful blast resistance gene [24]. *OsHIPP20* knockout lines did not show growth defect. Therefore, combined with *pi21*, *oshipp20* mutants may provide a novel source of durable resistance against blast disease. Knockout of *OsHIPP19*, in contrast to *OsHIPP20*, did not lead to an enhanced resistance against a compatible *M*. *oryzae* pathogen (**S13 Fig**). The expression level of *OsHIPP19* transcripts in non-induced leaves is twice as high as that of *OsHIPP20*, whereas the latter is more strongly induced than the former in suspension cultured cells after treatment with chitin (**Fig 1B**). Therefore, linking the lack of phenotype in knockout of *OsHIPP19* to its expression levels is not straightforward. Amino acid sequences of the C-terminal proline-rich domains of OsHIPP19 and OsHIPP20 are substantially different (**S16 Fig**), hinting their function may be different, i.e. by binding to different host proteins, which may possibly be the reason of different phenotypic outcomes of the knockout of the two genes. We need to clarify the functional differences between OsHIPP19 and OsHIPP20 in future studies. Also, we need to address the impact of *OsHIPP20* knockout for the infection of *M*. *oryzae* with AVR-Pik effector in future studies.

**Fig 5:**
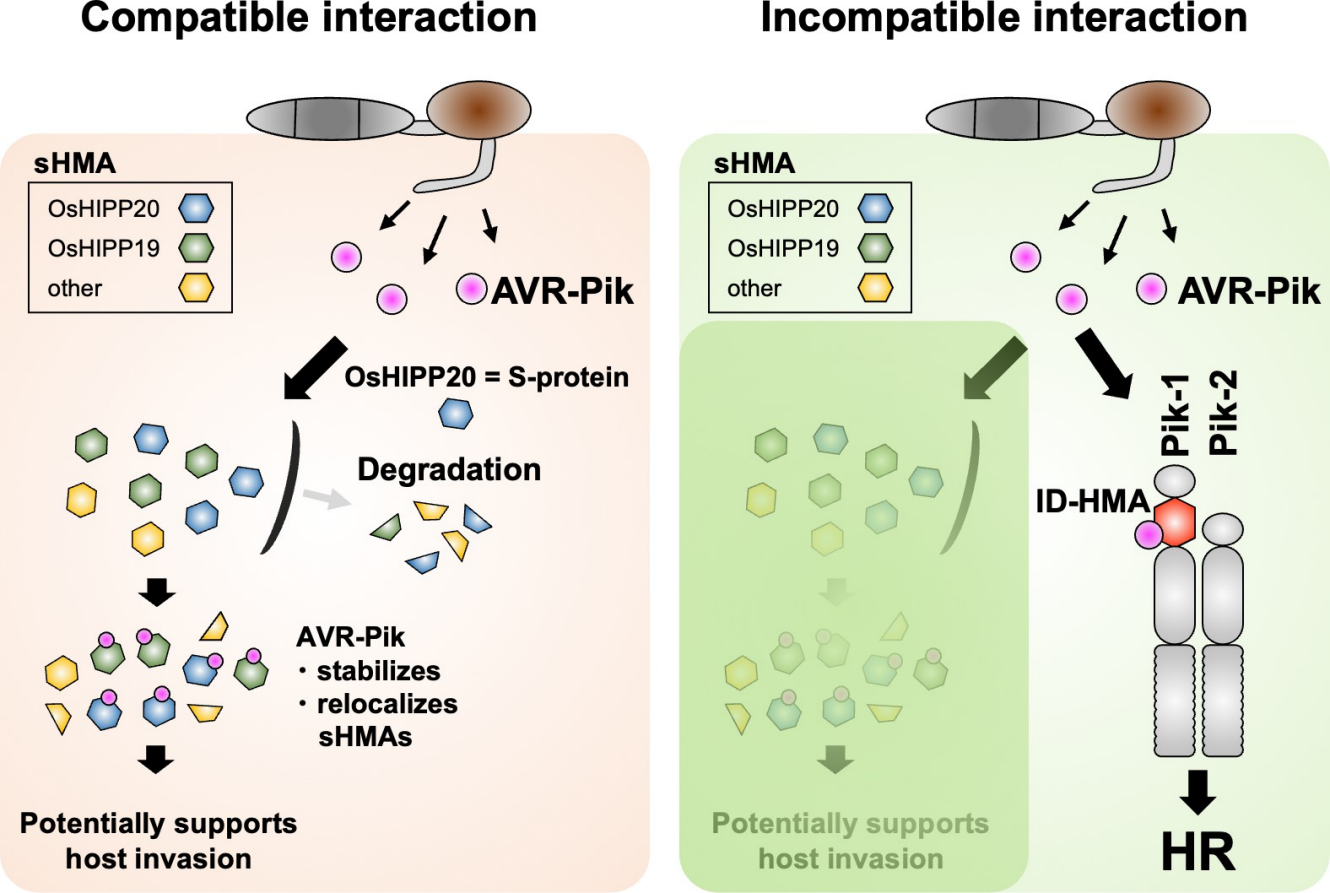
Schematic representation of a model showing molecular interactions between the AVR-Pik effector, rice sHMA proteins and Pik NLRs. In the compatible interaction (susceptible, left), AVR-Pik binds rice Clade A sHMA proteins, stabilizes and relocalizes them, possibly enhancing pathogen virulence. OsHIPP20 is a S-protein required for effective *M*. *oryzae* invasion. In the incompatible interaction (resistant, right), AVR-Pik interacts with integrated HMA domains of the Pik-1 NLRs which, together with Pik-2, triggers disease resistance by the hypersensitive response (HR). AVR-Pik and Pik seem involved in arms-race coevolution (selective force to enhancing interaction in Pik and evading interaction in AVR-Pik) by each generating multiple variants.

We also found that AVR-PikD binds and stabilizes OsHIPP19 and OsHIPP20 (**Fig 3**). We hypothesize that AVR-Pik–mediated stabilization of sHMA proteins suppresses host defenses, resulting in enhanced *M*. *oryzae* invasion of rice cells (**Fig 5**). Next, it will be important to determine the roles of the extended family of sHMA proteins in rice and other plants to understand the interplay between effector-mediated protein stabilization and disease.

Our conceptual and mechanistic understanding of how plant NLR proteins perceive pathogens continues to expand. A model termed the integrated domain hypothesis postulates that NLRs can bait pathogen effectors directly through integrated decoy/sensor domains [2–5]. These unconventional NLR domains are thought to have evolved by duplication and integration of an effector host target into the receptor protein. However, there are only few examples where the evolutionary origin of the NLR integrated domain could be traced to an effector target [9,47,48]. A recent study showed that AvrRPS4 effector of *Pseudomonas syringae* pv. pisi targets host WRKY54 to suppress immunity, but is recognized by an NLR RRS1 once bound by its WRKY-ID [49]. Here, we show that AVR-Pik interacts with sHMA proteins that belong to the same phylogenetic clade as the HMA domains integrated into the rice NLRs Pik-1 and RGA5. Therefore, throughout evolution, the Pik-1 NLR immune receptor has co-opted sHMA proteins through the integration of an HMA domain and neofunctionalization of this domain as a bait for the effector (**Fig 5**). This has launched a coevolutionary arms race between Pik-1 and AVR-Pik. Given that binding of AVR-Pik to Pik-1 HMA domains is necessary for triggering cell death and disease resistance in rice, new variants of AVR-Pik have arisen in *M*. *oryzae* populations that evade binding the integrated Pik-1 HMA but maintain their virulence activity [14,15,18,50]. Here we show that each of the AVR-Pik variants tested retain binding to OsHIPP19 and OsHIPP20 (**Fig 1D**), consistent with the view that stealthy effectors can retain virulence activities. This demonstrates that effector variation can affect the phenotypic outcomes of disease susceptibility and resistance independently through mediating bespoke interactions with different HMA domains. This elegant model highlights a surprisingly intimate relationship between plant disease susceptibility and resistance, as well as microbial virulence, driven by complex coevolutionary dynamics between pathogen and host.

## Materials and methods

### Construction of the maximum likelihood tree of HMA family genes

The protein sequences of the HMA domains were aligned by MAFFT [51] with the following method parameter set:—maxiterate 1000—localpair. Then, the maximum likelihood tree was constructed by IQ-TREE [52] with 1,000 bootstrap replicates [53]. The model was automatically selected by ModelFinder [54] in IQ-TREE [52].

### RNA-seq of rice leaves and suspension cultured cells and gene expression analysis

Total RNA was extracted from rice leaves and cultured cells (one hour after mock or chitin treatment) using SV Total RNA Isolation System (Promega, United States). One microgram of total RNA was used to prepare sequencing libraries with NEB Next Ultra II Directional RNA Library Prep Kit for Illumina (NEB, United States). These libraries were sequenced by paired-end (PE) sequencing using the Illumina Hiseq platform (Illumina, United States). The quality of the RNA-seq was evaluated using the FastQC (http://www.bioinformatics.babraham.ac.uk/projects/fastqc/). After QC, filtered reads were used for further analysis. Hisat2 [55] was used to align RNA-seq reads against the *O*. *sativa* reference genome downloaded from Rice Genome Annotation Project (http://rice.uga.edu/pub/data/Eukaryotic_Projects/o_sativa/annotation_dbs/pseudomolecules/). The levels of gene expression were scored by TPM (Transcripts Per Million) using StringTie [56].

### Yeast two-hybrid assay

To identify AVR-PikD–interacting proteins, signal peptide–truncated cDNA fragments of AVR-PikD were inserted into *Eco*RI and *Bam*HI sites of pGBKT7 (bait) vector (Takara Bio, Japan) to construct AVR-PikD/pGBKT7 (**S3 Table**). MATCHMAKER Library Construction & Screening kit (Takara Bio) was used to construct the rice cDNA library from leaf tissues of rice cultivar Sasanishiki 4, 24 and 48 h after inoculation with *M*. *oryzae* strain Sasa2 (race 037.1). Yeast strain AH109 competent cells were transformed with pGBKT7/AVR-PikD pGADT7-Rec and the rice cDNA library by using the polyethylene glycol/lithium acetate (PEG/LiAc) method, and plated on selective agar plates containing minimal medium without Trp, Leu, Ade and His, and supplemented with 20 mg/L of 5-Bromo-4-Chloro-3-indolyl a-D-galactopyranoside (X-α-Gal) and 10 mM 3-amino-1,2,4-triazole (3-AT). cDNAs in the library were transferred to pGAD-Rec vector harboring GAL4 activation domain (AD) by homologous recombination in yeast cells. Positive yeast transformants were streaked onto a minimal medium agar plate without Trp and Leu and used for sequence analysis.

To examine the protein–protein interactions between sHMAs and AVR-Pia, AVR-Pii, AVR1-CO39 and AVR-Pik alleles, yeast two-hybrid assay was performed as described previously [15]. Bait and prey plasmid vectors were constructed as described in **S3 Table**. Signal peptide–truncated cDNA fragments of AVRs were amplified by PCR by using primer set (**S3 Table**) and inserted into *Eco*RI and *Bam*HI sites of pGADT7 (prey) or pGBKT7 (bait) vectors (Takara Bio). sHMA cDNAs were synthesized from total RNAs of rice leaves (cultivar Sasanishiki) and inserted into pGADT7 and pGBKT7 by using *Spe*I and *Bam*HI sites as described in **S3 Table**. In the case of sHMAs containing *Spe*I or *Bam*HI site, In-Fusion HD Cloning Kit (Takara Bio) was utilized to construct plasmid vectors. The various combinations of bait and prey vectors were transformed into yeast strain AH109 by using the PEG/LiAc method. To detect the protein–protein interactions, ten-fold dilution series (×1, ×10^−1^, ×10^−2^) of yeast cells (×1: OD600 = 1.0) were spotted onto on basal medium lacking Trp, Leu, Ade and His but containing X-α-Gal (Takara Bio) and 10 mM 3-amino-1,2,4-triazole (3-AT). Positive signals were evaluated by blue coloration and growth of the diluted yeast. As a control, yeast growth on basal medium lacking Trp, Leu was also checked. Details of plasmids used are indicated in **S3 Table**. To check the protein accumulation in yeast cells, each transformant was propagated in the liquid basal medium lacking Trp, Leu with gentle shaking at 30°C overnight. Yeast cells from 10 ml medium were collected and 100 mg of yeast cells were treated with 400μl of 0.3 N NaOH for 15 min at room temperature. Resulting yeast extracts were used for western blot analysis using anti-Myc HRP-DirecT (MBL, Japan) for bait proteins and anti-HA (3F10)-HRP (Roche, Switzerland) for prey proteins.

### Plasmid construction for AlphaScreen

A total of 18 sHMAs belonging to CladeA and CladeB (Fig 1A and 1B) were amplified by PCR by using primer sets (**S3 Table**) and inserted into *Bsa*I sites of the level 0 vector pICH41308 (Addgene no. 47998) for the Golden Gate cloning [57]. FLAG-tagged sHMA was generated by Golden Gate assembly with pICH45089 (35S promoter (double), Addgene no. 50254), pAGT707 (5’ U-TMV+Ω, Addgene no. 51835), pICSL30005 (3xFLAG, Addgene no. 50299), pICH41308::sHMA and pICH41414 (3’UTR+ terminator, Addgene no. 50337) into a binary vector pICH47732 (Addgene no. 48000). Using pICH47732::sHMA as the PCR template, FLAG-tagged sHMA was amplified with forward primer (5’-CTACATCACCAAGATATCATGGATTATAAGGACCATGA-3’) and reverse primer (5’-TCTATACAAAACTAGTACTCACACATTATTATGGAG-3’), and then cloned into pEU-E01 vector [55] for wheat cell-free protein synthesis.

### Preparation of linier DNA templates for in vitro transcription and wheat cell-free protein synthesis

The *in vitro* transcription templates for the FLAG tagged sHMA were prepared by standard PCR with primer pairs (Spu, 5’- GCGTAGCATTTAGGTGACACT- 3’; SP-A1868, 5’- CCTGCGCTGGGAAGATAAAC -3’) using pEU-E01::sHMA expression plasmids as templates (as mentioned above). For preparation of *in vitro* transcription template for biotin-Myc tagged AVR-PikD was amplified by a slightly modified two-step PCR method [58] using pGBKT7::AVR-PikD plasmid as template. In the first round of PCR, the gene containing ORF and Myc-tagged sequence region was amplified by PCR using following primer pairs: AVR-PikD-S1, 5’- ccacccaccaccaccaATGGAGGAGCAGAAGCTGATCTC -3’(lower case letters indicate S1-linker sequence); AODA2306, 5’- AGCGTCAGACCCCGTAGAAA -3’. Next, a second round of PCR was carried out to add the SP6 promoter, the translation enhancer sequence E01, and the biotin ligation site sequence at the 5’ end of the ORF using the first PCR product as template. The following two sense primers and one antisense primer were used for PCR: Spu, 5’- GCGTAGCATTTAGGTGACACT -3’; deSP6E02-bls-S1, 5’- GGTGACACTATAGAACTCACCTATCTCTCTACACAAAACATTTCCCTACATACAACTTTCAACTTCCTATTATGGGCCTGAACGACATCTTCGAGGCCCAGAAGATCGAGTGGCACGAACTccacccaccaccaccaATG -3’ (underline and lower case letters indicate the biotin ligation site and S1-linker sequence, respectively); AODA2303, 5’- GTCAGACCCCGTAGAAAAGA -3’. All amplified PCR products were confirmed by agarose gel electrophoresis, and used for *in vitro* transcription and wheat cell-free protein synthesis.

### Cell-free protein synthesis

In vitro transcription and wheat cell-free protein synthesis were performed using WEPRO7240 expression kit (Cell-Free Sciences, Japan). Transcript was made from each of the DNA templates mentioned above using the SP6 RNA polymerase. The translation reaction was performed in the bilayer mode [59] using WEPRO7240 expression kit (Cell-Free Sciences) according to the manufacture’s instruction. For biotin labeling, 1 μl of crude biotin ligase (BirA) produced by the wheat cell-free expression system was added to the bottom layer, and 0.5 μM (final concentration) of d-biotin (Nacalai Tesque, Japan) was added to both upper and bottom layers, as described previously [60]. The aliquots were used for the expression analysis and protein-protein interaction assay.

### Protein-protein interaction assays using AlphaScreen

The protein-protein interaction (PPI) between biotinylated AVR-PikD and FLAG-tagged sHMA proteins were detected with AlphaScreen technology provided by PerkinElmer. PPI assays were carried out in a total volume of 15 μl containing 1 μl of biotinylated AVR-PikD, and 1 μl of FLAG-sHMA proteins in the AlphaScreen buffer (100 mM Tris-HCl (pH8.0), 0.01% Tween20, 1mg/ml BSA) at 25°C for 1 h in a 384-well Optiplate (PerkinElmer, San José, USA). In accordance with the AlphaScreen IgG (ProteinA) detection kit (PerkinElmer) instruction manual, 10 μl of detection mixture containing AlphaScreen buffer, 5 μg/mL anti-DYKDDDDK monoclonal antibody (clone 1E6, FUJIFILM Wako, Japan), 0.1 μl of streptavidin-coated donor beads, and 0.1 μl of Protein A-coated acceptor beads were added to each well of the 384-well Optiplate, followed by incubation at 25°C for 1 h. Luminescence was analyzed using the AlphaScreen detection program using EnSight Multimode Plate Reader (PerkinElmer). All data represent the average of three independent experiments and the background was controlled using a dihydrofolate reductase (DHFR) from *E*. *coli*.

### Binding complex modeling by ColabFold

For the prediction of AVR-PikD/sHMA complex structures, we used the alphafold2advanced.ipynb notebook (ColabFold_v1.5.2) [30] (https://colab.research.google.com/github/sokrypton/ColabFold/blob/v1.5.2/AlphaFold2.ipynb, accessed on August to October 2023) with the default mode. Display of the predicted AVR-PikD/sHMA complex and measurement of interatomic distances were performed using Waals (Altif Laboratories Inc., Tokyo, Japan). Multimer confidence score is calculated as AlphaFold-multimer score (0.8*ipTM+0.2*pTM) in **S5 and S6 Figs** [61].

### Generation of rice mutants of *OsHIPP*s by CRISPR/Cas9 system

Rice plants with mutated *OsHIPP19*, *OsHIPP20* or *OsHPP04* were generated using the CRISPR/Cas9 system developed by Mikami et al. [62] Primers OsHIPP19_gRNA1-F: 5’-gttgAAGCTGGTGGTGATGGCCTC-3’ and OsHIPP19_gRNA1-R: 5’-aaacGAGGCCATCACCACCAGCTT-3’ were annealed and cloned into pU6::ccdB::gRNA cloning vector by digestion with BbsI as the target sequence. The target sequence with the OsU6 promoter was replaced into the pZH::gYSA::MMCas9 vector by digestion with *Asc*I and *Pac*I, generating pZH::gYSA::MMCas9::OsHIPP19_gRNA1. Binary vector, pZH::gYSA::MMCas9:: OsHIPP19_gRNA2, pZH::gYSA::MMCas9::OsHIPP20_gRNA1, pZH::gYSA::MMCas9::OsHIPP20_gRNA2 and pZH::gYSA::MMCas9:: OsHPP04_gRNA1 were constructed by the same method; details of the primers are listed in **S3 Table**. The rice cultivar ‘Sasanishiki’ was used for Agrobacterium-mediated transformation following the methods of Toki et al. [63]. Thereafter, regenerated T0 plants were sequenced using primers listed in **S3 Table** and the mutation type was analyzed.

### Growth conditions of plants and *M*. *oryzae*

In this study, rice plants were grown in a closed greenhouse at 28°C. *M*. *oryzae* strains Sasa2 and Ken53-33 were grown on oatmeal-agar medium (40 g/L oatmeal, 5 g/L sucrose, 20 g/L agar) at 25°C for 10 days. Conidium formation was induced for 4 days at 28°C under dark-blue light for rice pathogenicity assays.

### Rice pathogenicity assays

Rice leaf blade punch inoculations were performed using the *M*. *oryzae* strains Sasa2 (without AVR-Pik alleles). A conidial suspension (3 × 10^5^ conidia/mL) was inoculated onto punched area of leaves one month after sowing. The inoculated plants were placed in a dew chamber at 27°C for 24 h in the dark and transferred to a growth chamber with a photoperiod of 16 h. Disease lesions were scanned 10 days post-inoculation(dpi) and the lesion size was measured using ‘Image J’ software [64]. For spray inoculation assay, rice leaves of 6 weeks after sowing were spray-inoculated with the *M*. *oryzae* isolate Ken53-33. The concentration of conidial suspension was adjusted to 5 × 10^5^ conidia/mL with 0.01% Tween-20 (v/v). Samples were collected at 4 dpi and extracted gDNA by NucleoSpin Plant II kit (MACHEREY-NAGEL, Germany). We determined the ratio of *M*. *oryzae* actin gene (XM_003719823.1) to rice ACTIN gene (Os03g61970.1) by quantitative PCR [65].

### Transient protein expression assay in *N*. *benthamiana*

For transient protein expression, *Agrobacterium tumefaciens* strain GV3101 was transformed with the relevant binary constructs (**S3 Table**). To detect sHMA (OsHIPP19 and OsHIPP20) protein accumulation and stability in *N*. *benthamiana*, several combinations of *Agrobacterium* transformants (the ratio of each transformant is 2:7:1 for sHMA: AVR (GUS): P19; final concentration is OD_600_ = 1.0) were infiltrated using a needleless syringe into leaves of 3- to 4-weeks-old *N*. *benthamiana* plants grown at 23°C in a greenhouse. Two days after infiltration, leaves were collected and homogenized by using a Multi-Beads Shocker (Yasui-Kikai, Japan) under cooling with liquid nitrogen. Then 2 ml of extraction buffer (10% glycerol, 25 mM Tris–HCl pH 7.0, 10 mM DTT, 1 tablet / 50ml cOmplete Protease Inhibitor Cocktail [Roche, Switzerland]) was added to 1 mg of leaf tissues and further homogenized. After centrifugation at 20,000×g for 15 min, the supernatant was collected and the pellet was resuspended in 2 ml (the same as supernatant volume) of extraction buffer. The supernatants and pellet samples were subjected to SDS-PAGE followed by western blotting. Proteins were immunologically detected by using anti-HA (3F10)-HRP (Roche, Switzerland), anti-Myc-tag (HRP-DirecT) (MBL, Japan) and Anti-β-Glucuronidase (N-Terminal) antibodies produced in rabbit (Sigma-Aldrich, United States). The luminescent images were detected by luminescent Image Analyzer LAS-4000 (Cytiva, Japan) after treatment of ChemiLumi One Super or Ultra (Nacalai Tesque, Japan).

### Co-immunoprecipitation assay

For co-immunoprecipitation assays, Myc-tagged sHMA (eGFP) and HA-tagged AVR-PikD binary constructs (**S3 Table**) were transformed into *Agrobacterium tumefaciens* strain GV3101. Proteins were co-expressed in *N*. *benthamiana* and extracted from the leaves (approximately 150 mg) with 400 mL of extraction buffer (50 mM Tris-HCl pH 7.5 and 150 mM NaCl). Extracts were incubated with Anti-Myc-tag mAb-Magnetic Agarose (MBL, Japan) at 4°C for 1 h. Myc-agarose was washed with the same buffer 3 times and bound protein was eluted with 1x SDS sample buffer. The eluates were used for western blot analysis using anti-HA (3F10)-HRP (Roche, Switzerland) and anti-Myc-tag (HRP-DirecT) (MBL) antibodies.

### Localization of OsHIPP19 and OsHIPP20

To visualize subcellular localization of OsHIPP17, OsHIPP19 and OsHIPP20, N-terminally GFP-tagged OsHIPP17, OsHIPP19, OsHIPP20 expression constructs were generated by Golden Gate assembly with pICH45089, pAGT707, pICH41531 (GFP, Addgene no. 50321), pICH41308::OsHIPP17 (::OsHIPP19,:: OsHIPP20) and pICH41414 into a binary vector pICH47732. These constructs were transformed into *Agrobacterium tumefaciens* strain GV3101 for transient expression in *N*. *benthamiana* (the ratio of each transformant is 2:7:1 for GFP: AVR (GUS): P19; final concentration is OD_600_ = 1.0). To visualize plasmodesmata, Aniline blue fluorochrome (Biosupplies, Australia) was used at 0.01 mg/ml and infiltrated into the leaves 2 days after agroinfiltration before being analyzed by confocal microscopy [31]. The fluorescence was visualized by a Nikon AX Confocal Microscope System (Nikon, Japan). Aniline blue fluorochrome was excited using 405 nm laser and captured at 460–480 nm. GFP was excited using a 488 nm laser and captured at 505–555 nm (Fig 3B).

For studying subcellular localization of OsHIPP19 and OsHIPP20 in Fig 3C, pCambia::3xMyc-eGFP-OsHIPP19 and pCambia::3xMyc-eGFP-OsHIPP20 were transformed into *Agrobacterium tumefaciens* strain GV3101 for transient expression in *N*. *benthamiana* (the ratio of each transformant is 2:7:1 for GFP: AVR (GUS): P19; final concentration is OD_600_ = 1.0). GFP fluorescence in the leaves 2 days after agroinfiltration were observed by an Olympus FluoView FV1000-D confocal laser scanning microscope (Olympus). GFP was excited with an HeNe(G) laser. A DM488/543/633 diachronic mirror, SDM beam splitter, and BA505-525 emission filter were used for observation.

## Supporting information

S1 Fig AVR-PikD interacts with Clade A sHMA proteins in yeast 2-hybrid assay (Y2H). **(A)** Interactions between AVR-PikD and a subset of sHMA proteins were tested by Y2H. sHMA proteins were used as prey and AVR-PikD as bait (left panels) and AVR-PikD was used as prey and sHMA proteins as bait (right panels). Results with the conditions of stringent selection (QDO+3AT: SD/-Trp/-Leu/-Ade/-His, X-α-Gal,10mM 3AT) as well as no selection (DDO: SD/-Trp/-Leu) are shown. **(B)** Interactions between empty vector products and a subset of sHMA proteins were tested by Y2H. sHMA proteins were used as prey and empty vector product as bait (left panels) and empty vector product was used as prey and sHMA proteins as bait (right panels). Results with the conditions of stringent selection (QDO+3AT) as well as no selection (DDO) are shown. **(C)** Western blot analysis confirms protein production in the Y2H experiment shown in **S1A Fig**. The bait protein was tagged with the Myc epitope and the prey protein was tagged with the HA epitope. The bands of proteins expressed from the constructs are marked by red asterisks. The positions of molecular size marker are indicated in the right (kDa).
(TIFF)

S2 Fig Interaction between AVR-PikD and Clade A sHMA proteins as addressed by AlphaScreen. **(A)** AVR-PikD and sHMA proteins were produced by wheat germ translation system, and were subjected to AlphaScreen interaction assay. The values indicate relative AlphaScreen signal (AS) as compared to that of AVR-PikD / OsHIPP19. The error bars represent SD of 3 replications. **(B)** Western blot analysis confirms protein production in the AlphaScreen as shown in **S2A Fig**. The sHMA proteins were tagged with the FLAG epitope and detected by anti-FLAG antibody. The synthesized protein bands are marked by red asterisks. The positions of molecular size marker are indicated in the right (kDa).
(TIFF)

S3 Fig AVR-Pia and AVR1-CO39 do no bind Clade A sHMAs or Pi21. Four *M*. *oryzae* effectors, AVR-Pia, AVR-PikD, AVR-Pii and AVR1-CO39 were tested for their binding with Clade A sHMAs (OsHIPP19, OsHIPP20, OsHPP04, OsHPP03 and LOC_Os04g39380) as well as Pi21 (OsHIPP05) of Clade B in Y2H assay with high stringency condition (QDO+3AT) as well as no selection (DDO). The HMA domain of Pikm-1 NLR protein (Pikm-HMA) interacts with AVR-PikD (Kanzaki et al. 2012) [15] and used as a positive control. AVR-Pii was used as a negative control. (**A)** shows the results when effectors were used as bait and sHMAs as prey. **(B)** shows the results when effectors were used as prey and sHMAs as bait. **(C)** Western blot results corresponding to Y2H in **S3A Fig**. Pikm-1-HMA as well as sHMAs (prey) were detected by anti-HA antibody (left panel), whereas AVRs (bait) were detected by an anti-Myc antibody (right panel). **(D)** Western blot results corresponding to Y2H in **S3B Fig**. Pikm-1-HMA as well as sHMAs (bait) were detected by anti-Myc antibody (left), whereas AVRs (prey) were detected by an anti-HA antibody (right). The protein bands expressed from the constructs were marked by red asterisks. Molecular sizes (kDa) are indicated in the right of panels.
(TIFF)

S4 Fig Y2H results with empty vector indicate no autoactivation with the AVR-Pik effector constructs. **(A)** Results of high stringency selection (QDO+3AT) as well as no selection (DDO) are shown. **(BC)** Results of Western blot analysis confirming AVR-Pik-alleles (A, C, D, E) and OsHIPP19 and OsHIPP20 protein production in Y2H experiment as shown in **Fig 1D**. OsHIPP proteins were used as prey and AVR-Pik alleles as bait **(B)**. OsHIPP proteins were used as bait and AVR-Pik alleles as prey **(C)**. The bands of proteins expressed from the constructs are marked by red asterisks. The positions of molecular size marker are indicated in the right (kDa).
(TIFF)

S5 Fig AVR-PikD/sHMA complex as predicted by ColabFold_v1.5.2. **(A)** Predicted complex structure of AVR-PikD and sHMA that were shown to interact in Y2H (**Fig 1**). **(B)** Predicted complex structure of AVR-PikD and sHMA that were not shown to interact in Y2H (**Fig 1**). For each protein, predicted binding structure (top), pLDDT (bottom left), predicted aligned error (bottom right) are shown. Predicted regions with low pLDDT score (< 50) are not displayed. AlphaFold (AF) -multimer score = 0.8*ipTM + 0.2*pTM (Yin et al. 2022) [61]. **(C)** Five types of AVR-PikD (light green) / sHMA (white or orange) complexes (Type A to E) predicted by ColabFold. Predicted complex structure of AVR-PikD and sHMA that were shown to interact in Y2H (**Fig 1**) all belonged to Type A, while those of AVR-PikD and sHMA non-interacting in Y2H belonged to either of Type B, C, D, E.
(TIFF)

S6 Fig AVR-PikD/sHMA (OsHIPP16_D68DEL, OsHIPP16_S80K, OsHIPP16_D68DEL_S80K) complex predictions as generated by ColabFold_v1.5.2. For each protein, predicted binding structure (top), pLDDT (bottom left), predicted aligned error (bottom right) are shown. Predicted regions with low pLDDT score (< 50) are not displayed. AlphaFold (AF) -multimer score = 0.8*ipTM + 0.2*pTM (62).
(TIFF)

S7 Fig Western blot analysis confirms protein production in Y2H of Fig 2C. **(A)** Y2H interactions between the variants of OsHIPP16 (OsHIPP16, OsHIPP16_D68DEL, OsHIPP16_S80K, OsHIPP16_D68DEL_S80K) and the empty vector products. **(BC)** Results of Western blot analysis confirming AVR-PikD and OsHIPP16 variant protein production in Y2H experiment as shown in **Fig 2C**. OsHIPP16 variant proteins were used as prey and AVR-Pik as bait **(B:** left panel**)**. OsHIPP16 variant proteins were used as bait and AVR-Pik as prey **(B:** right panel**)**. OsHIPP16 variant proteins were used as prey and empty as bait **(C:** left panel**)**. OsHIPP16 variant proteins were used as bait and empty as prey **(C:** right panel**)**. **(D)** Western blot analysis confirms protein production in the AlphaScreen as shown in **Fig 2D**. The sHMA proteins were tagged with the FLAG epitope and detected by anti-FLAG antibody. The bands of proteins expressed from the constructs are marked by red asterisks. The positions of molecular size marker are indicated in the right (kDa).
(TIFF)

S8 Fig A mutant version of OsHPP02 binds AVR-PikD. **(A)** AVR-PikD/sHMA (OsHPP02_D79V) complex predictions were generated in ColabFold_v1.5.2. Predicted binding structure (top), pLDDT (bottom left), predicted aligned error (bottom right) are shown. Predicted regions with low pLDDT score (< 50) are not displayed. AlphaFold (AF) -multimer score = 0.8*ipTM + 0.2*pTM (Yin et al. 2022) [61]. **(B)** Y2H interaction assay between AVR-PikD and OsHPP02 and OsHPP02_D79V. **(C)** Western blot analysis confirms protein production in Y2H as shown in **S8B Fig. (D)** AlphaScreen interaction assay between AVR-PikD and OsHPP02 and OsHPP02_D79V. Relative signal strength as compared to that between OsHIPP02 and DHFR (negative control) is given. The error bars represent SD of 3 replications. **(E)** Western blot analysis confirms protein production in the AlphaScreen as shown in **S8D Fig**. The sHMA proteins were tagged with the FLAG epitope and detected by anti-FLAG antibody. The bands of proteins expressed from the constructs are marked by red asterisks. The positions of molecular size marker are indicated on the right (kDa).
(TIFF)

S9 Fig AVR-PikD stabilizes sHMAs in planta. **(A)** The results for pellet fraction after fractionation of leaf extract are shown. AVR-PikD seems to accumulate in the pellet fraction when it does not bind sHMA. **(B)** Western blots showing the protein bands corresponding to the N-terminally Myc-tagged proteins Myc-OsHIPP19 (top) and Myc-OsHIPP20 (bottom) from the supernatant fraction (S) and the pellet fraction (P) of *Nicotiana benthamiana* leaf extract. Myc-OsHIPP19 or Myc-OsHIPP20 protein was transiently expressed in *N*. *benthamiana* leaf either with no protein (empty), HA:AVR-Pii or AVR-Pik-D:HA protein.
(TIFF)

S10 Fig Co-immunoprecipitation experiment shows AVR-PikD does not bind OsHIPP17. Binding assay between OsHIPP17 and AVR-PikD. Epitope-tagged proteins, AVR-PikD:HA and FLAG:OsHIPP17 were expressed in *Nicotiana benthamiana* leaves. The leaf extract was applied to an anti-FLAG antibody column and the bound proteins were detected by an anti-FLAG antibody (top) and an anti-HA antibody (bottom). AVR-PikD:HA band detected in the anti-HA blot after co-immunoprecipitation is caused by non-specific weak binding of AVR-PikD:HA to anti-FLAG antibody column.
(TIFF)

S11 Fig Supplement to Fig 3B. Subcellular localization of OsHIPPs (GFP:OsHIPP19, GFP:OsHIPP20 and GFP:OsHIPP17) expressed in *N*. *benthamiana* leaves in the presence of GUS, HA:AVR-Pii and AVR-PikD:HA. Scale bar: 20 μm.
(TIFF)

S12 Fig AVR-Pik-D binding affects subcellular localization of sHMAs expressed in *N*. *benthamiana*. **(A)** Results of western blot analysis of proteins expressed in *N*. *benthamiana* leaves as shown in **Fig 3B**. **(B)** A full-size image of the western blot as shown in (A). **(C)**Histograms showing the number of GFP:OsHIPP19 and GFP:OsHIPP20 puncta structure in *N*. *benthamiana* cells in the presence of GUS, HA:AVR-Pii and AVR-PikD:HA.
(TIFF)

S13 Fig Knockout of *OsHIPP19* and *OsHPP04* does not alter susceptibility of rice against a compatible *M*. *oryzae* isolate. **(A)** A compatible *M*. *oryzae* isolate Sasa2 was punch inoculated onto the leaves of rice cultivar Sasanishiki as well as the sHMA-knockout lines of Sasanishiki (oshipp19#1, oshipp19#2 and oshpp04). Box plots show lesion area sizes in the rice lines (top). Statistical significance is shown after Wilcoxon rank sum test. Photos of typical lesions developed on the leaves after inoculation of *M*. *oryzae* (bottom). The number of leaves used for experiments are indicated below. **(B)** A table showing guide RNA and transgenic line nomenclature (left), location of guide RNA used for CRISPR/Cas9 mutagenesis (center) and the resulting nucleotide changes (center and right). PAM is indicated with blue and the sgRNA sequence is indicated with red letters.
(TIFF)

S14 Fig **(A)** Results of spray inoculation of *M*. *oryzae* Ken53-33 to the wild-type Sasanishiki (WT) and two OsHIPP20-knockout lines g1-2 and g2-1 (2^nd^ replication). Statistical significance is shown after Wilcoxon rank sum test. **(B)** Images of leaves of Sasanishiki wild-type (WT) and OsHIPP20 knockout lines *g1-2* and *g2-1* 4 days after spray inoculation of *M*. *oryzae* Ken53-33.
(TIFF)

S15 Fig A Sasanishiki line (g2-3) in which both *OsHIPP20* and *LOC_Os04g32290*.*1* gene were knocked out shows enhanced resistance against a compatible *M*. *oryzae* but does not show growth defect. **(A)** Punch inoculation results of T2 generation of homozygous KO rice plants. Statistical significance is shown after Wilcoxon rank sum test. **(B)** No growth defect in *OsHIPP20* + *LOC_Os04g32290*.*1* KO line as compared to the wild type. Top: Overview of the W/W and m/m plants. Bottom: Box plot showing culm length (left), panicle length (center) and panicle number (right) of W/W and m/m plants. The numbers (***N***) below the graph show the number of individuals for which measurement was made. Statistical significance is shown after Wilcoxon rank sum test.
(TIFF)

S16 Fig Amino acid sequence alignment of OsHIPP19 and OsHIPP20. HMA domain and proline rich domain (PRD) are indicated by blue and red square, respectively.
(TIFF)

S1 Table Rice proteins that interacted with AVR-PikD in the Y2H screen. We carried out two Y2H screens (1st and 2nd). The number of positive clones with insert sequences corresponding to the designated proteins are shown.
(XLSX)

S2 Table Summary of whole genome sequence comparison between the wild type Sasanishiki (WT) and *OsHIPP20* KO lines. (XLSX)

S3 Table A summary of constructs used in this study. (XLSX)

S4 Table Raw data. (XLSX)
