# Supplementary figures and images for: The blast pathogen effector AVR-Pik binds and stabilizes rice heavy metal-associated (HMA) proteins to co-opt their function in immunity
Source: PLoS Pathog. 2024 Nov 18;20(11):e1012647. doi: 10.1371/journal.ppat.1012647 (PMC11611257; doi:10.1371/journal.ppat.1012647)

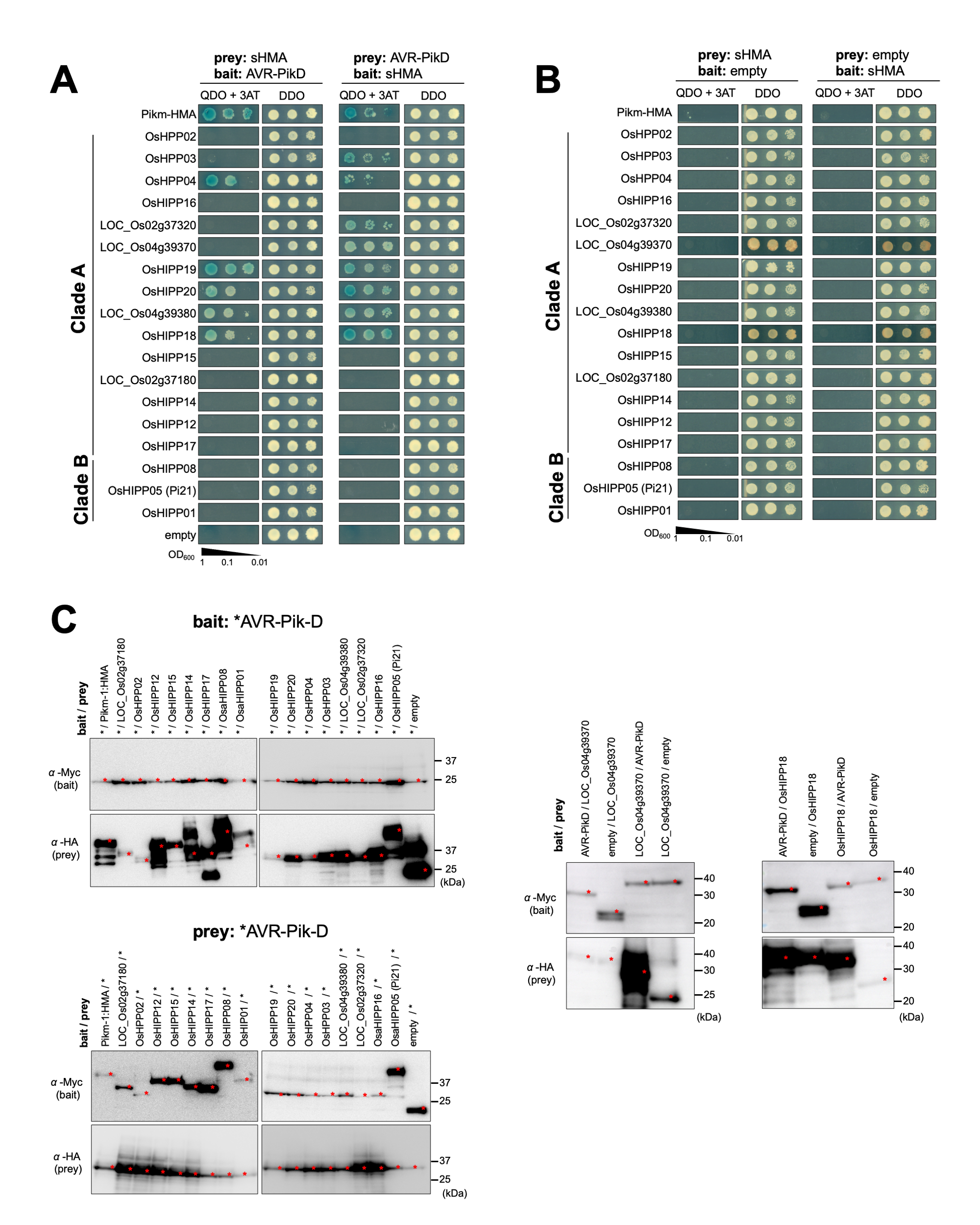

Supplement: S1 Fig — (A) Interactions between AVR-PikD and a subset of sHMA proteins were tested by Y2H. sHMA proteins were used as prey and AVR-PikD as bait (left panels) and AVR-PikD was used as prey and sHMA proteins as bait (right panels). Results with the conditions of stringent selection (QDO+3AT: SD/-Trp/-Leu/-Ade/-His, X-α-Gal,10mM 3AT) as well as no selection (DDO: SD/-Trp/-Leu) are shown. (B) Interactions between empty vector products and a subset of sHMA proteins were tested by Y2H. sHMA proteins were used as prey and empty vector product as bait (left panels) and empty vector product was used as prey and sHMA proteins as bait (right panels). Results with the conditions of stringent selection (QDO+3AT) as well as no selection (DDO) are shown. (C) Western blot analysis confirms protein production in the Y2H experiment shown in S1A Fig. The bait protein was tagged with the Myc epitope and the prey protein was tagged with the HA epitope. The bands of proteins expressed from the constructs are marked by red asterisks. The positions of molecular size marker are indicated in the right (kDa). (TIFF) [file ppat.1012647.s001.tiff]

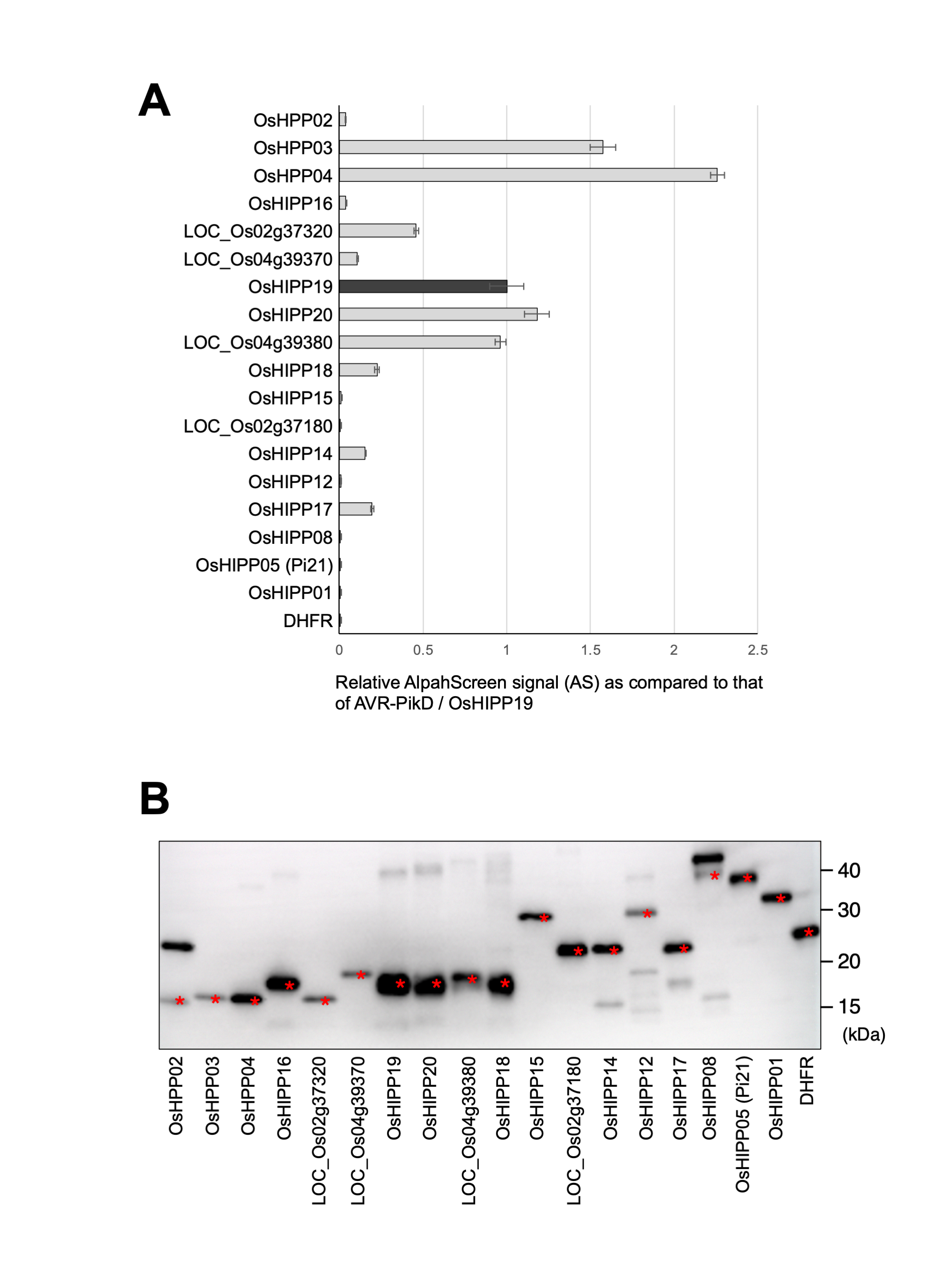

Supplement: S2 Fig — (A) AVR-PikD and sHMA proteins were produced by wheat germ translation system, and were subjected to AlphaScreen interaction assay. The values indicate relative AlphaScreen signal (AS) as compared to that of AVR-PikD / OsHIPP19. The error bars represent SD of 3 replications. (B) Western blot analysis confirms protein production in the AlphaScreen as shown in S2A Fig. The sHMA proteins were tagged with the FLAG epitope and detected by anti-FLAG antibody. The synthesized protein bands are marked by red asterisks. The positions of molecular size marker are indicated in the right (kDa). (TIFF) [file ppat.1012647.s002.tiff]

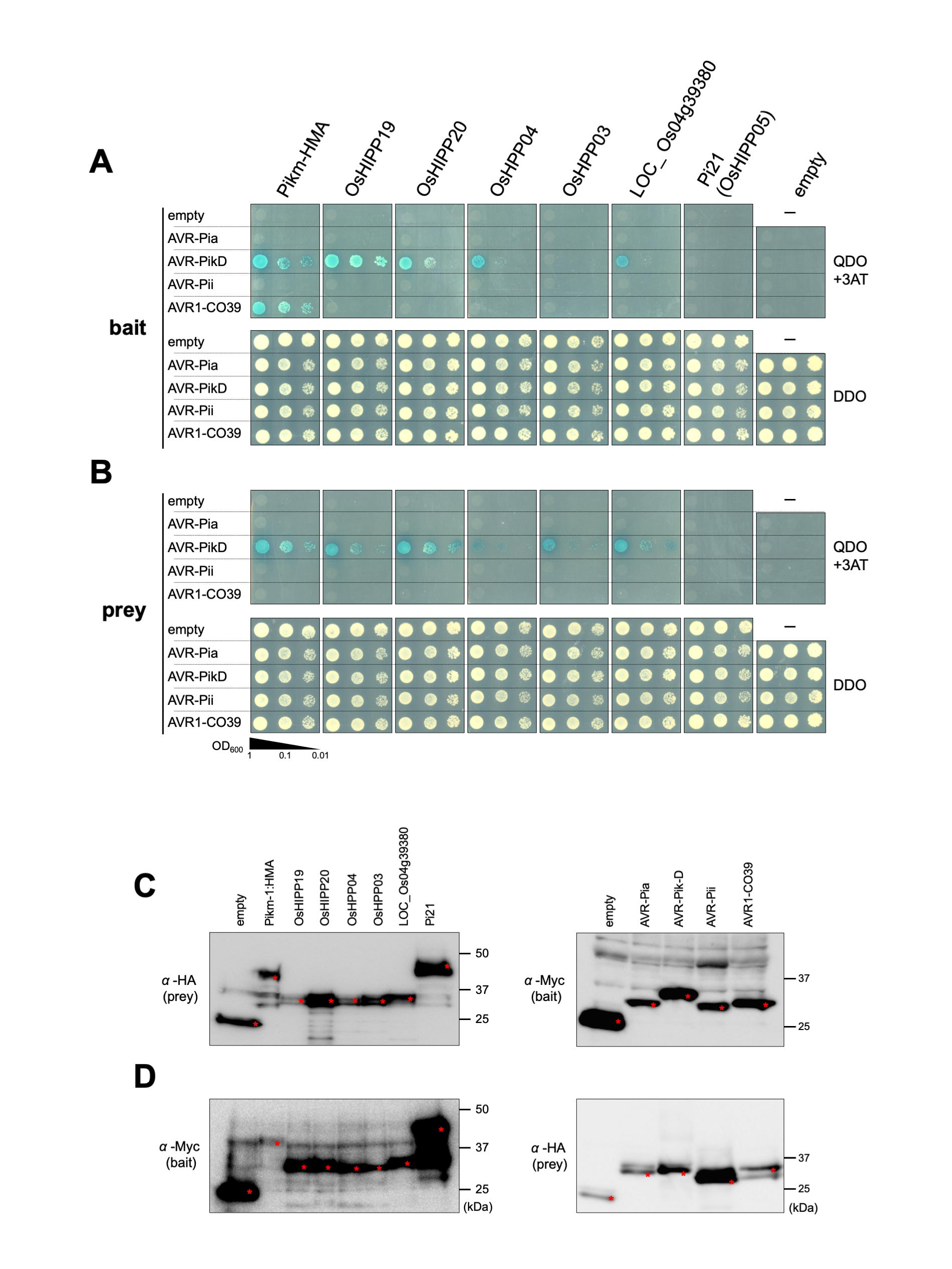

Supplement: S3 Fig — Four M. oryzae effectors, AVR-Pia, AVR-PikD, AVR-Pii and AVR1-CO39 were tested for their binding with Clade A sHMAs (OsHIPP19, OsHIPP20, OsHPP04, OsHPP03 and LOC_Os04g39380) as well as Pi21 (OsHIPP05) of Clade B in Y2H assay with high stringency condition (QDO+3AT) as well as no selection (DDO). The HMA domain of Pikm-1 NLR protein (Pikm-HMA) interacts with AVR-PikD (Kanzaki et al. 2012) [15] and used as a positive control. AVR-Pii was used as a negative control. (A) shows the results when effectors were used as bait and sHMAs as prey. (B) shows the results when effectors were used as prey and sHMAs as bait. (C) Western blot results corresponding to Y2H in S3A Fig. Pikm-1-HMA as well as sHMAs (prey) were detected by anti-HA antibody (left panel), whereas AVRs (bait) were detected by an anti-Myc antibody (right panel). (D) Western blot results corresponding to Y2H in S3B Fig. Pikm-1-HMA as well as sHMAs (bait) were detected by anti-Myc antibody (left), whereas AVRs (prey) were detected by an anti-HA antibody (right). The protein bands expressed from the constructs were marked by red asterisks. Molecular sizes (kDa) are indicated in the right of panels. (TIFF) [file ppat.1012647.s003.tiff]

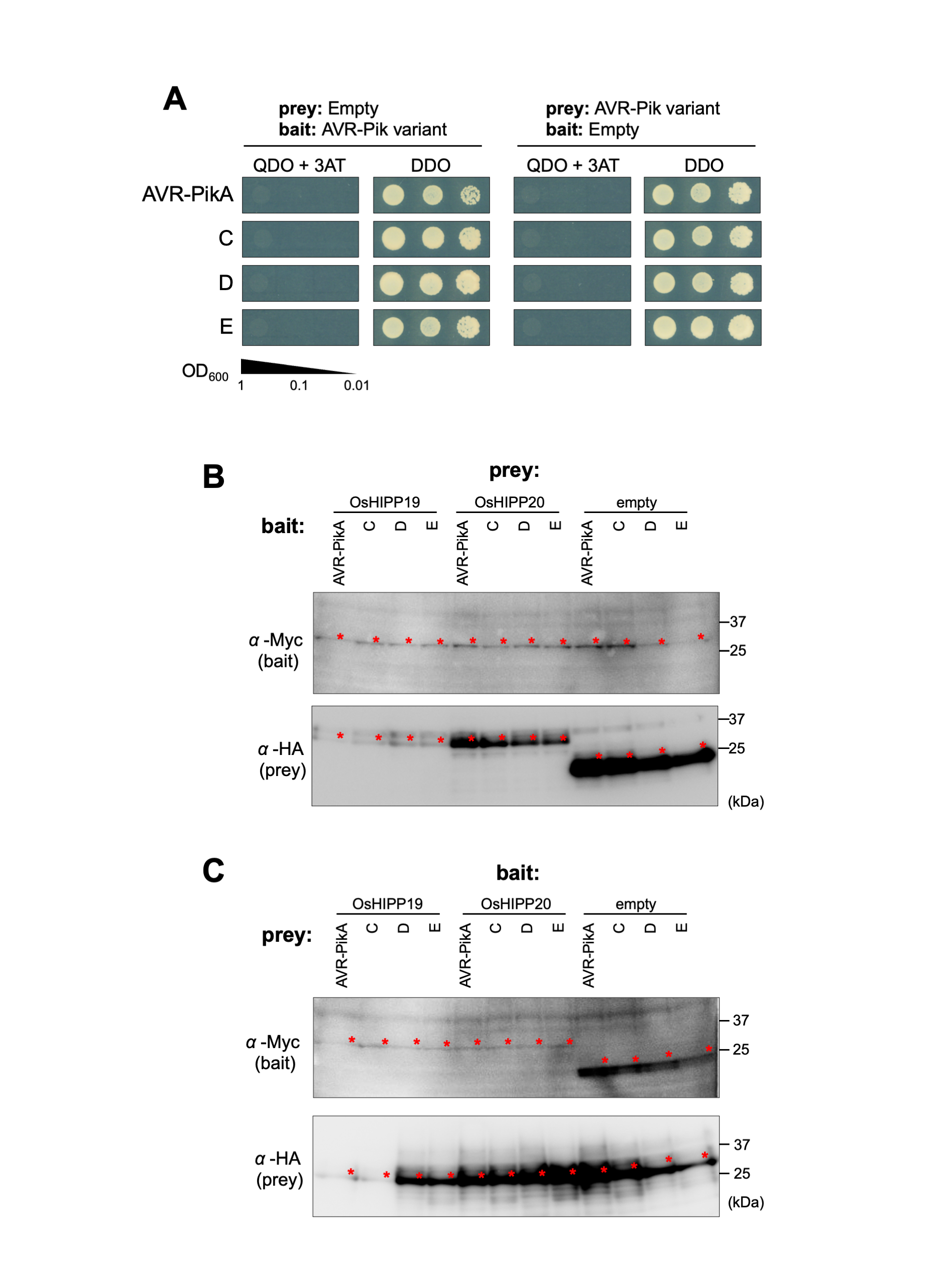

Supplement: S4 Fig — (A) Results of high stringency selection (QDO+3AT) as well as no selection (DDO) are shown. (BC) Results of Western blot analysis confirming AVR-Pik-alleles (A, C, D, E) and OsHIPP19 and OsHIPP20 protein production in Y2H experiment as shown in Fig 1D. OsHIPP proteins were used as prey and AVR-Pik alleles as bait (B). OsHIPP proteins were used as bait and AVR-Pik alleles as prey (C). The bands of proteins expressed from the constructs are marked by red asterisks. The positions of molecular size marker are indicated in the right (kDa). (TIFF) [file ppat.1012647.s004.tiff]

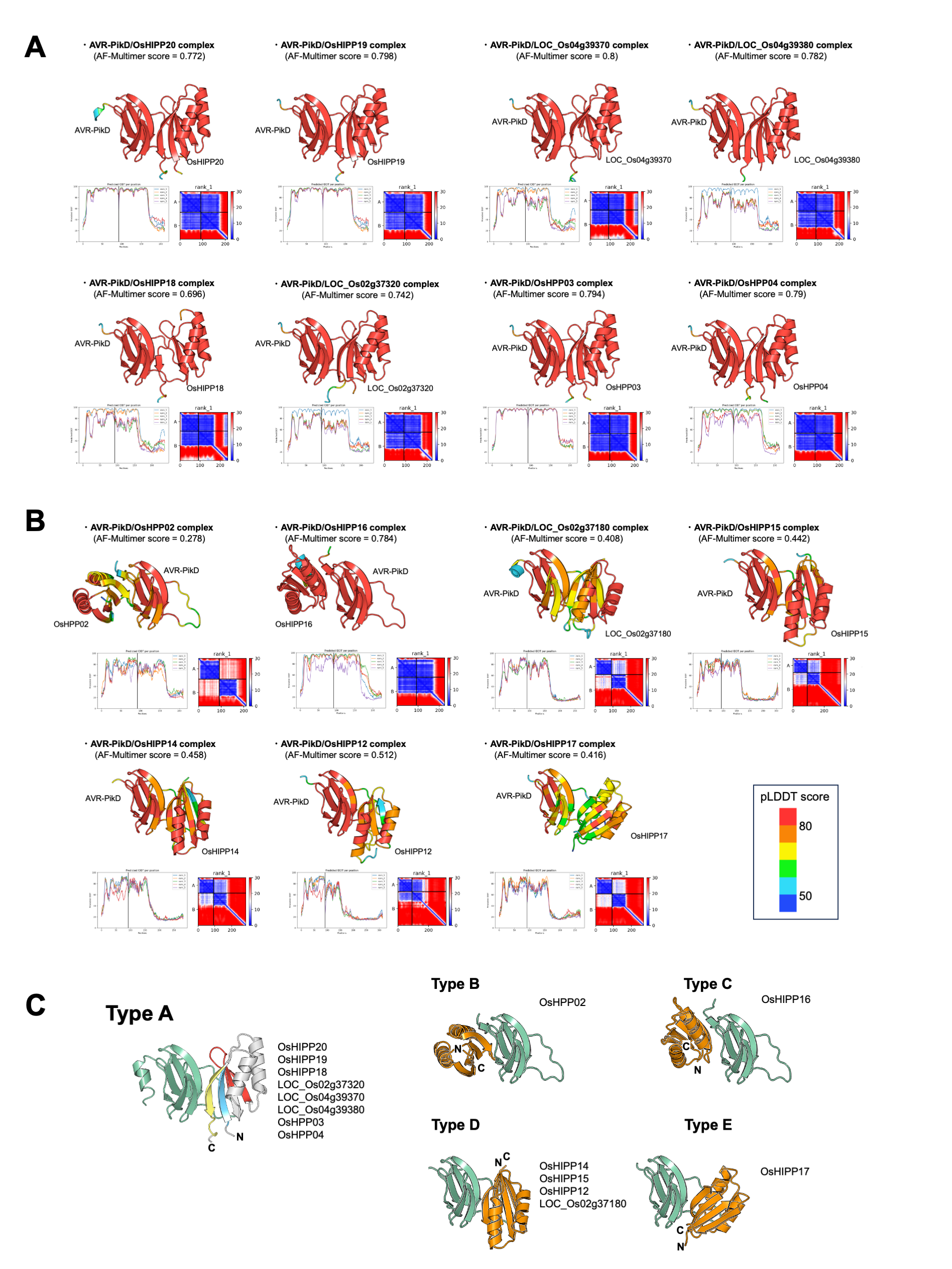

Supplement: S5 Fig — (A) Predicted complex structure of AVR-PikD and sHMA that were shown to interact in Y2H (Fig 1). (B) Predicted complex structure of AVR-PikD and sHMA that were not shown to interact in Y2H (Fig 1). For each protein, predicted binding structure (top), pLDDT (bottom left), predicted aligned error (bottom right) are shown. Predicted regions with low pLDDT score (< 50) are not displayed. AlphaFold (AF) -multimer score = 0.8*ipTM + 0.2*pTM (Yin et al. 2022) [61]. (C) Five types of AVR-PikD (light green) / sHMA (white or orange) complexes (Type A to E) predicted by ColabFold. Predicted complex structure of AVR-PikD and sHMA that were shown to interact in Y2H (Fig 1) all belonged to Type A, while those of AVR-PikD and sHMA non-interacting in Y2H belonged to either of Type B, C, D, E. (TIFF) [file ppat.1012647.s005.tiff]

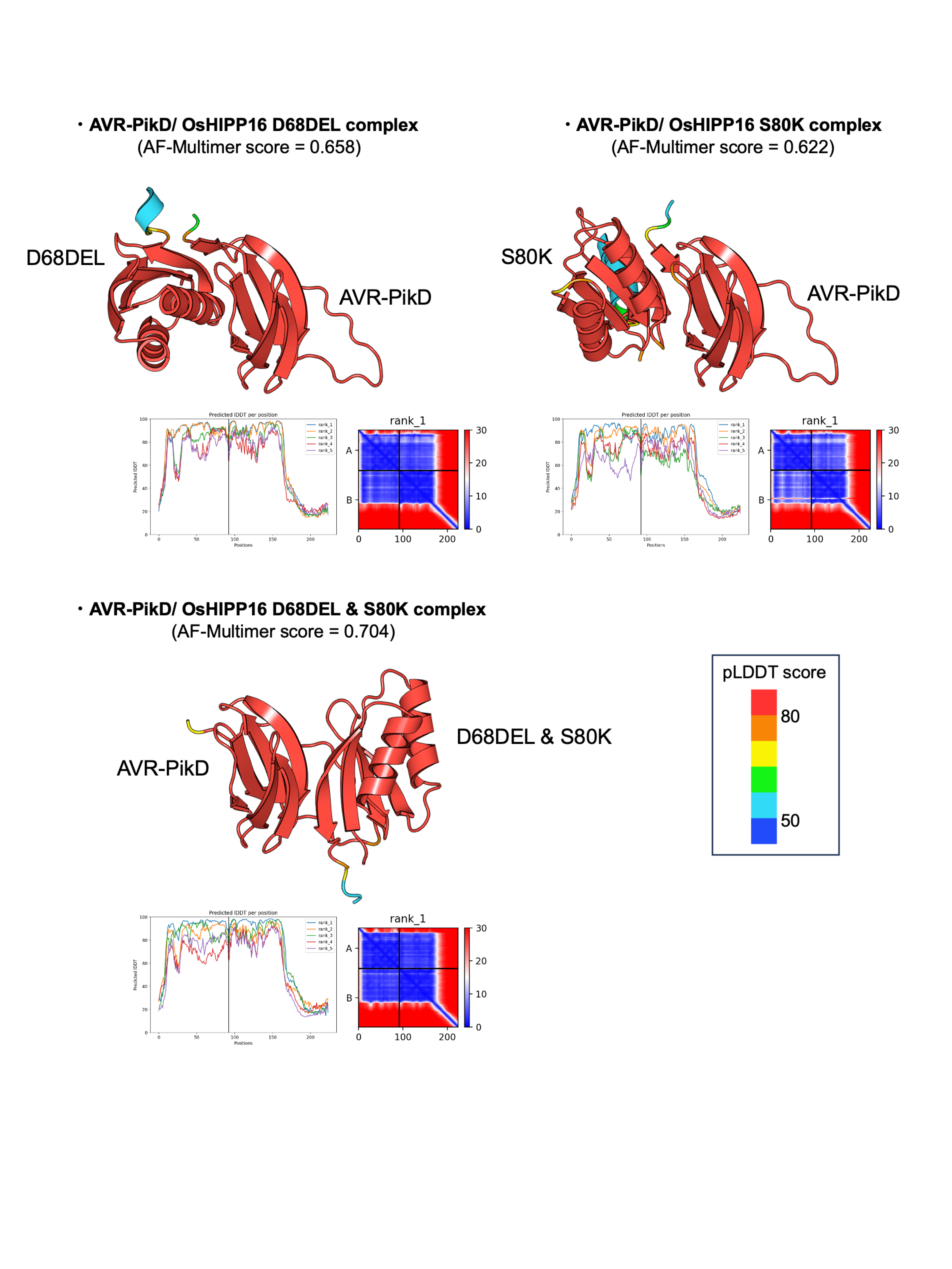

Supplement: S6 Fig — For each protein, predicted binding structure (top), pLDDT (bottom left), predicted aligned error (bottom right) are shown. Predicted regions with low pLDDT score (< 50) are not displayed. AlphaFold (AF) -multimer score = 0.8*ipTM + 0.2*pTM (62). (TIFF) [file ppat.1012647.s006.tiff]

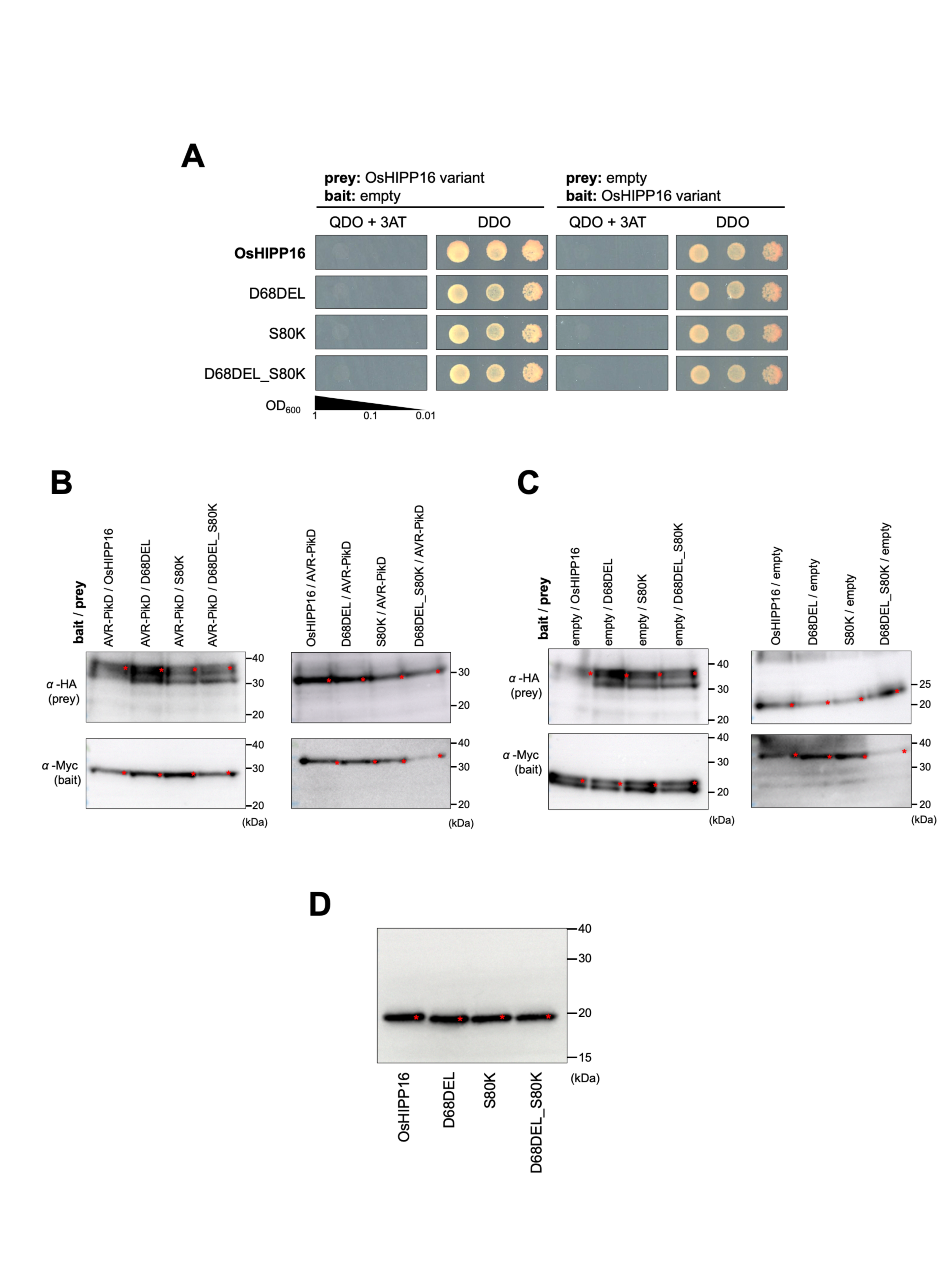

Supplement: S7 Fig — (A) Y2H interactions between the variants of OsHIPP16 (OsHIPP16, OsHIPP16_D68DEL, OsHIPP16_S80K, OsHIPP16_D68DEL_S80K) and the empty vector products. (BC) Results of Western blot analysis confirming AVR-PikD and OsHIPP16 variant protein production in Y2H experiment as shown in Fig 2C. OsHIPP16 variant proteins were used as prey and AVR-Pik as bait (B: left panel). OsHIPP16 variant proteins were used as bait and AVR-Pik as prey (B: right panel). OsHIPP16 variant proteins were used as prey and empty as bait (C: left panel). OsHIPP16 variant proteins were used as bait and empty as prey (C: right panel). (D) Western blot analysis confirms protein production in the AlphaScreen as shown in Fig 2D. The sHMA proteins were tagged with the FLAG epitope and detected by anti-FLAG antibody. The bands of proteins expressed from the constructs are marked by red asterisks. The positions of molecular size marker are indicated in the right (kDa). (TIFF) [file ppat.1012647.s007.tiff]

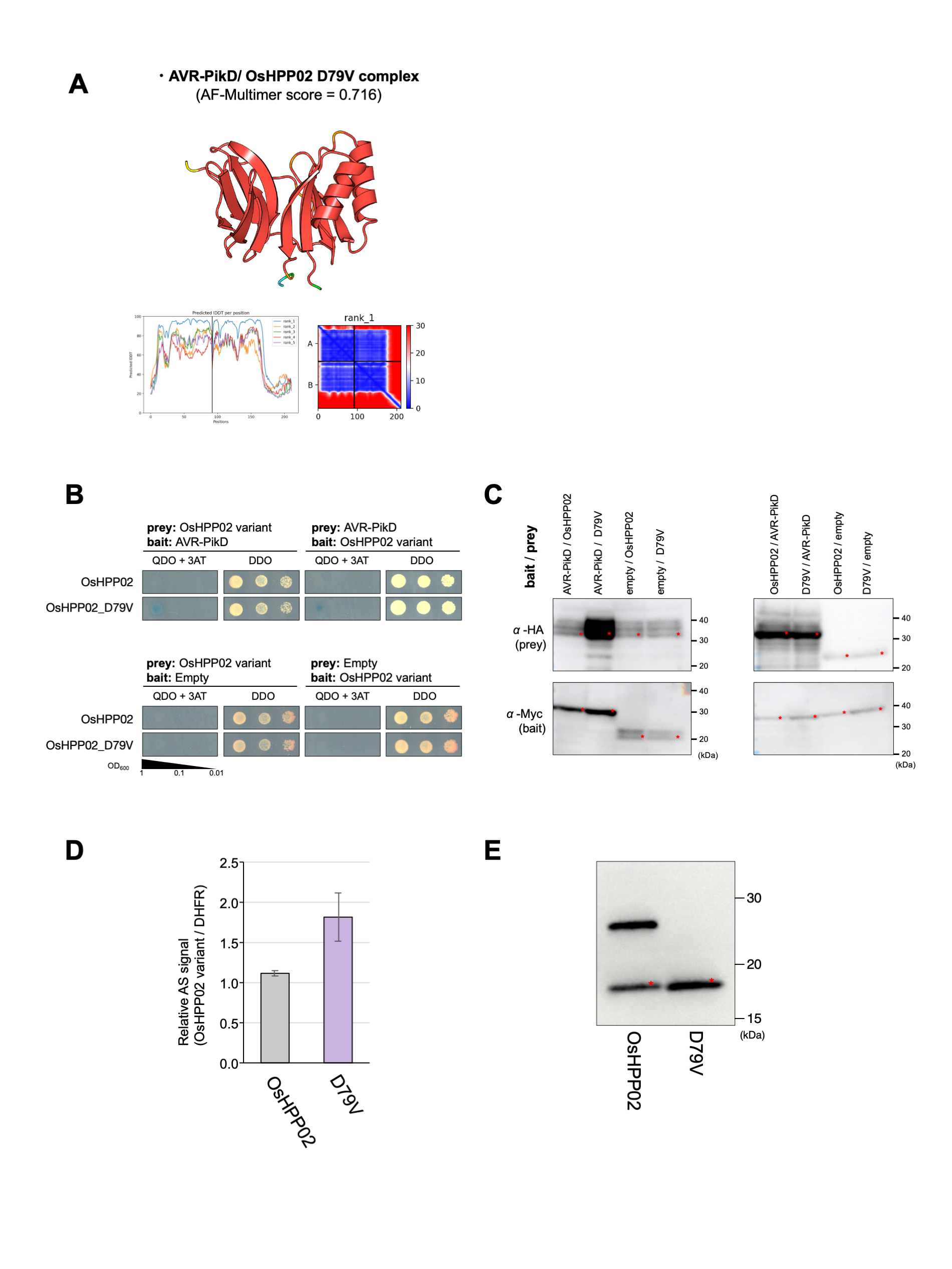

Supplement: S8 Fig — (A) AVR-PikD/sHMA (OsHPP02_D79V) complex predictions were generated in ColabFold_v1.5.2. Predicted binding structure (top), pLDDT (bottom left), predicted aligned error (bottom right) are shown. Predicted regions with low pLDDT score (< 50) are not displayed. AlphaFold (AF) -multimer score = 0.8*ipTM + 0.2*pTM (Yin et al. 2022) [61]. (B) Y2H interaction assay between AVR-PikD and OsHPP02 and OsHPP02_D79V. (C) Western blot analysis confirms protein production in Y2H as shown in S8B Fig. (D) AlphaScreen interaction assay between AVR-PikD and OsHPP02 and OsHPP02_D79V. Relative signal strength as compared to that between OsHIPP02 and DHFR (negative control) is given. The error bars represent SD of 3 replications. (E) Western blot analysis confirms protein production in the AlphaScreen as shown in S8D Fig. The sHMA proteins were tagged with the FLAG epitope and detected by anti-FLAG antibody. The bands of proteins expressed from the constructs are marked by red asterisks. The positions of molecular size marker are indicated on the right (kDa). (TIFF) [file ppat.1012647.s008.tiff]

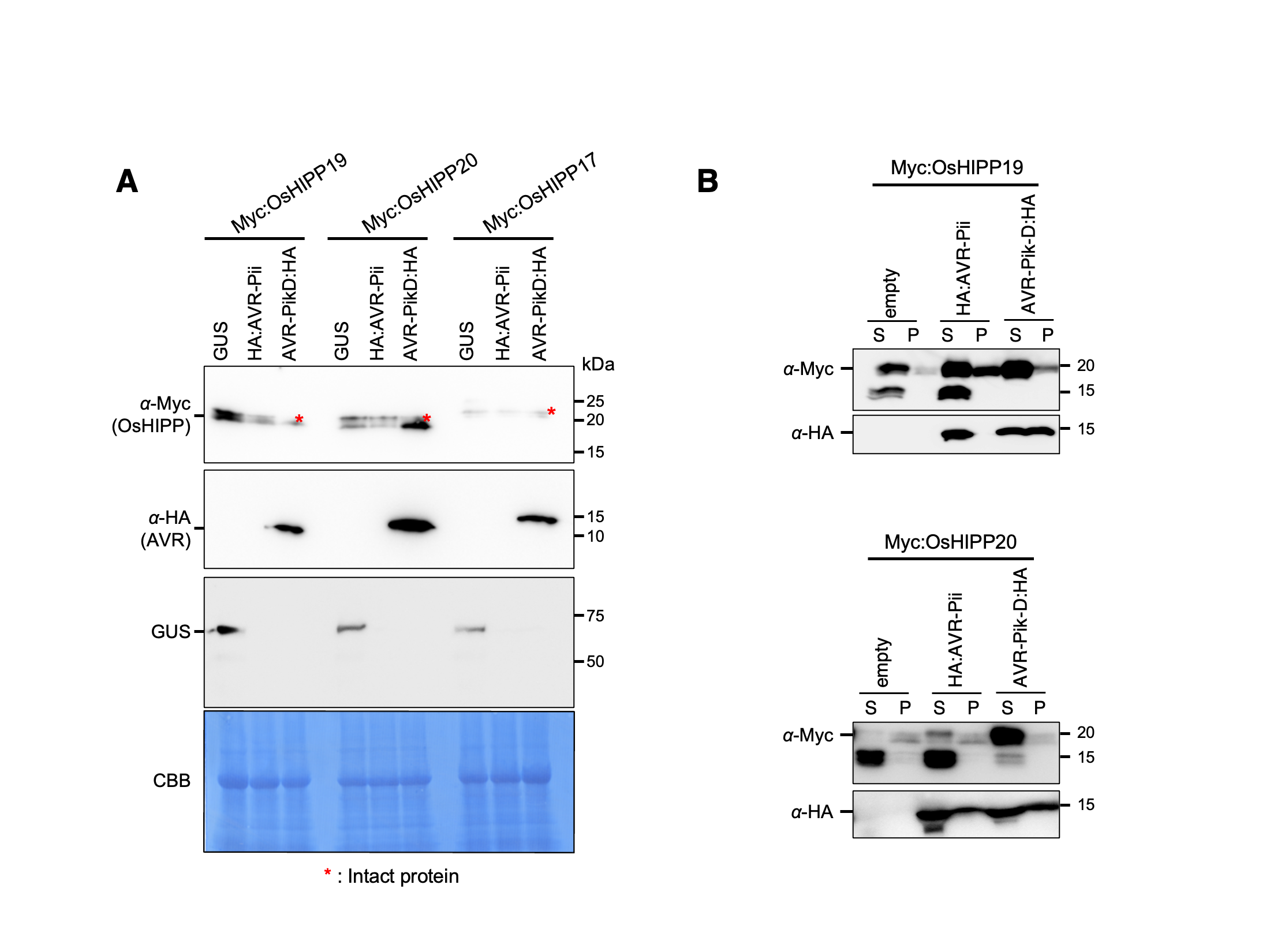

Supplement: S9 Fig — (A) The results for pellet fraction after fractionation of leaf extract are shown. AVR-PikD seems to accumulate in the pellet fraction when it does not bind sHMA. (B) Western blots showing the protein bands corresponding to the N-terminally Myc-tagged proteins Myc-OsHIPP19 (top) and Myc-OsHIPP20 (bottom) from the supernatant fraction (S) and the pellet fraction (P) of Nicotiana benthamiana leaf extract. Myc-OsHIPP19 or Myc-OsHIPP20 protein was transiently expressed in N. benthamiana leaf either with no protein (empty), HA:AVR-Pii or AVR-Pik-D:HA protein. (TIFF) [file ppat.1012647.s009.tiff]

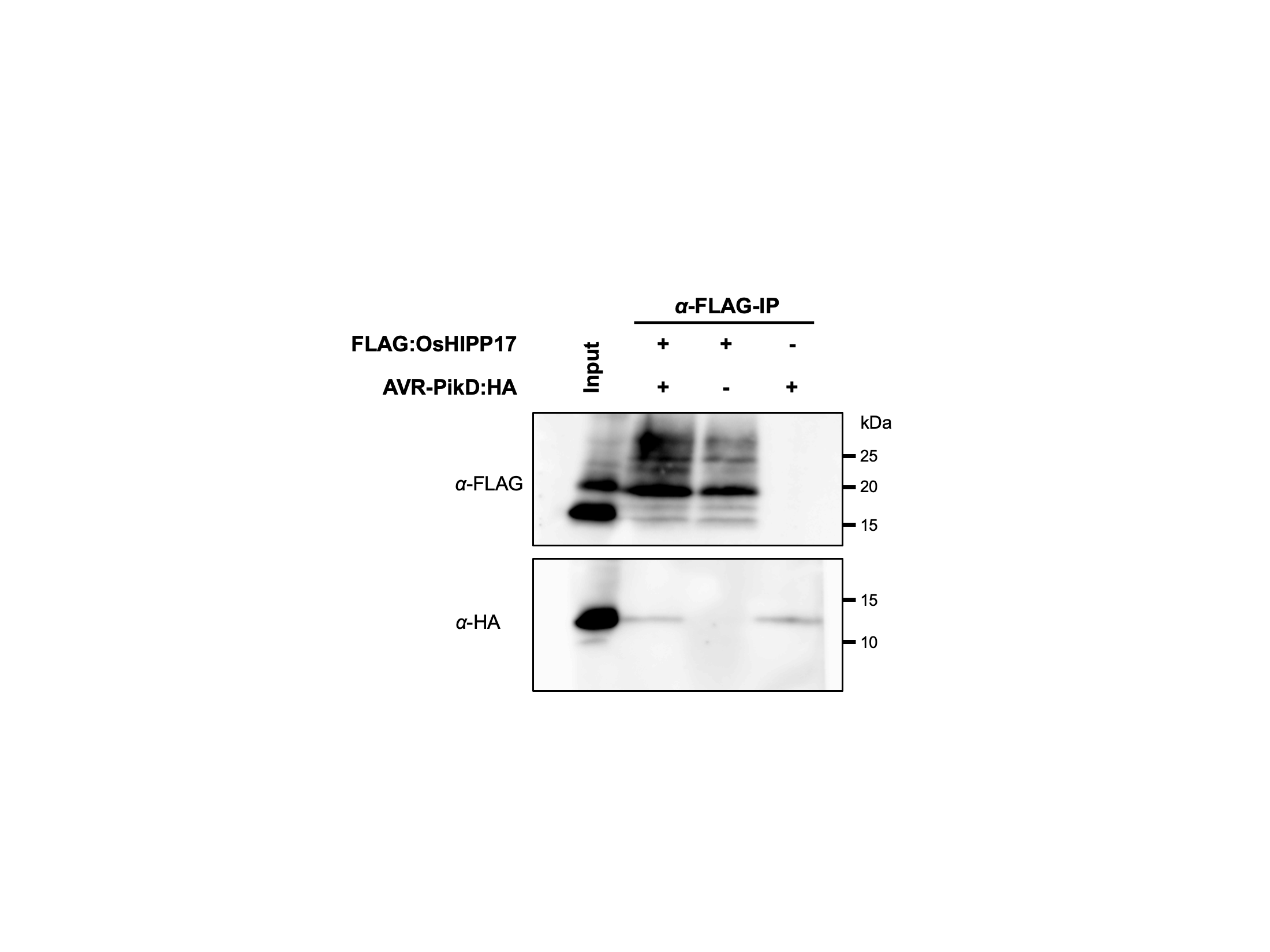

Supplement: S10 Fig — Binding assay between OsHIPP17 and AVR-PikD. Epitope-tagged proteins, AVR-PikD:HA and FLAG:OsHIPP17 were expressed in Nicotiana benthamiana leaves. The leaf extract was applied to an anti-FLAG antibody column and the bound proteins were detected by an anti-FLAG antibody (top) and an anti-HA antibody (bottom). AVR-PikD:HA band detected in the anti-HA blot after co-immunoprecipitation is caused by non-specific weak binding of AVR-PikD:HA to anti-FLAG antibody column. (TIFF) [file ppat.1012647.s010.tiff]

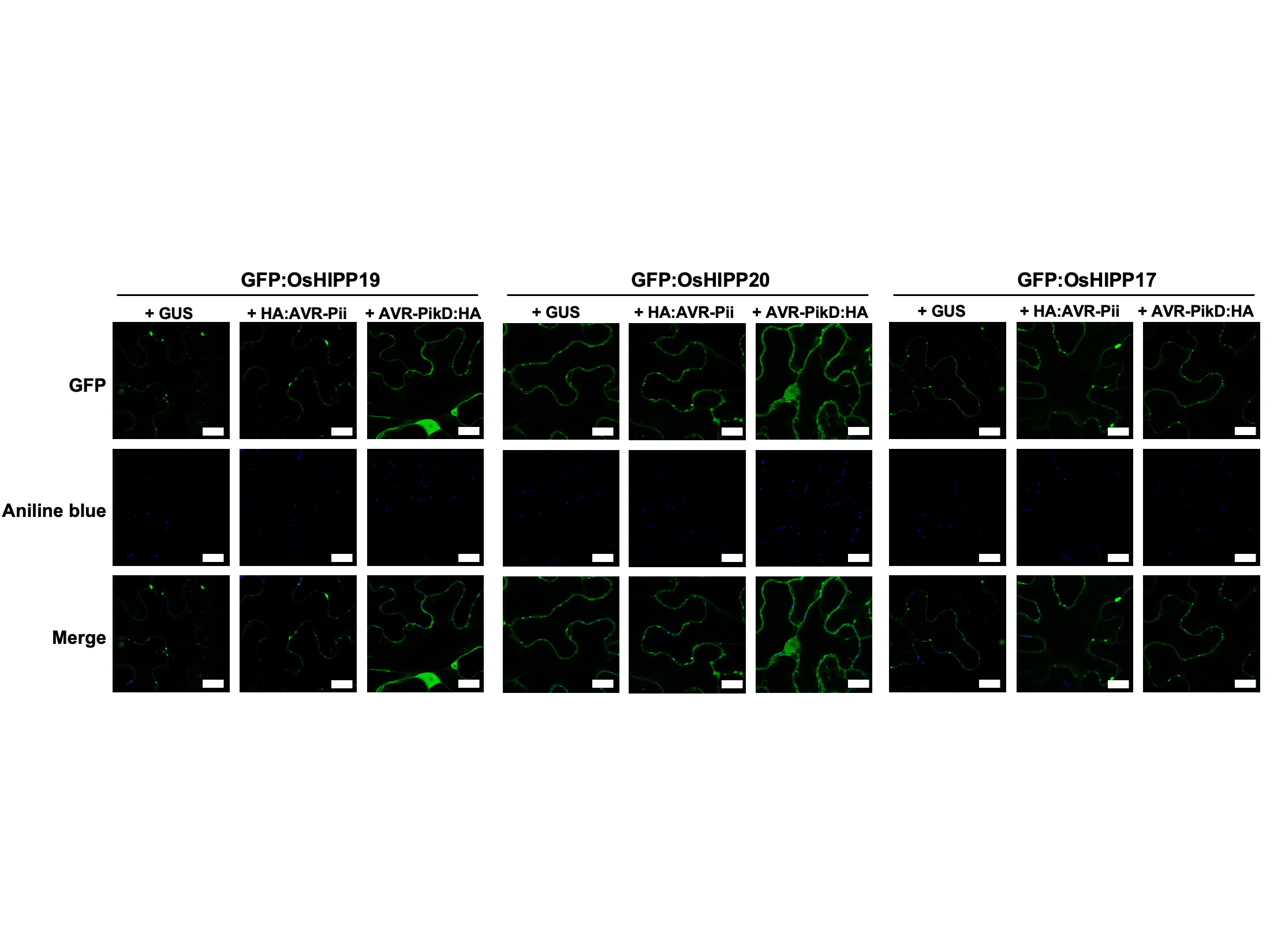

Supplement: S11 Fig — Subcellular localization of OsHIPPs (GFP:OsHIPP19, GFP:OsHIPP20 and GFP:OsHIPP17) expressed in N. benthamiana leaves in the presence of GUS, HA:AVR-Pii and AVR-PikD:HA. Scale bar: 20 μm. (TIFF) [file ppat.1012647.s011.tiff]

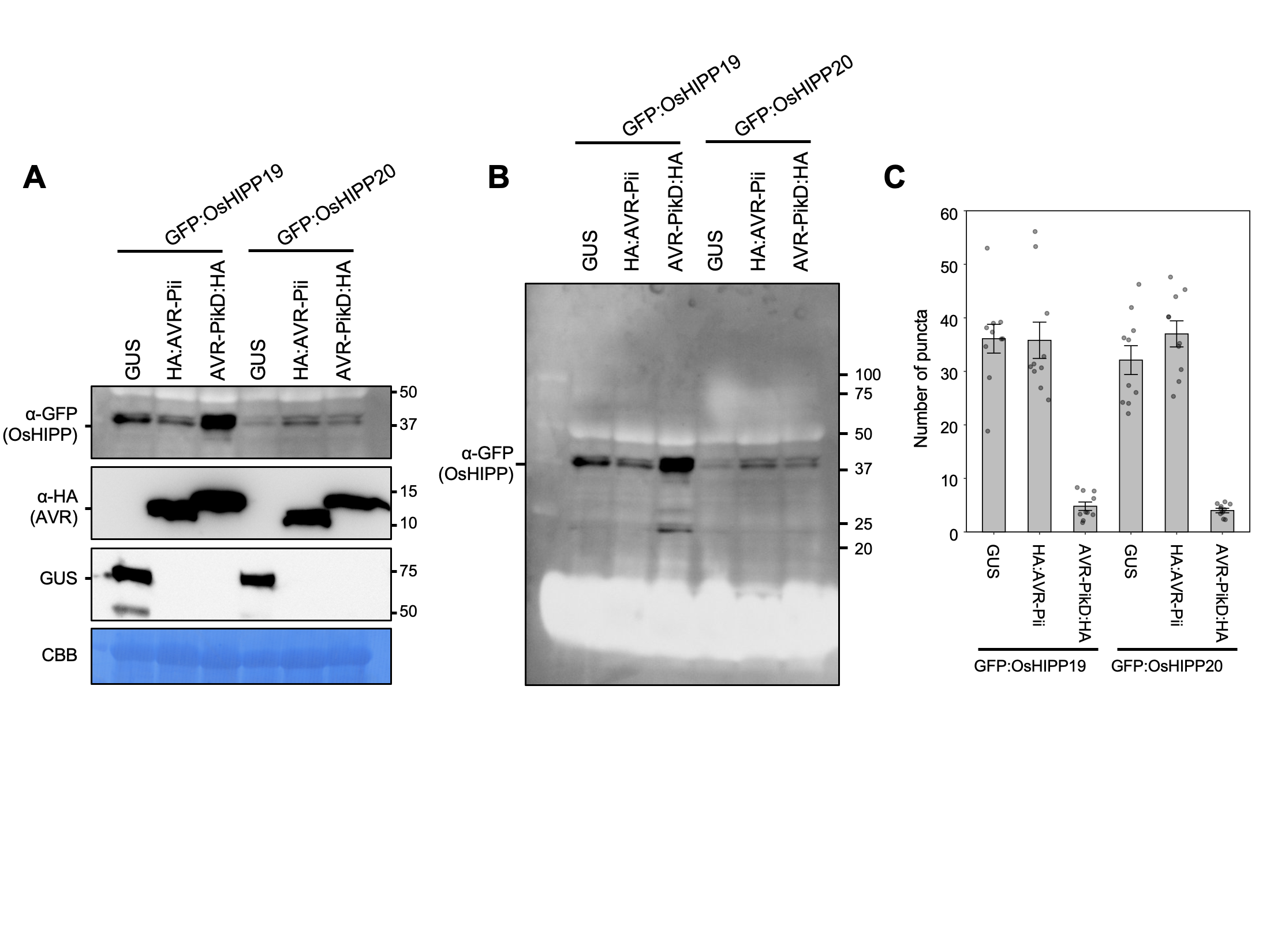

Supplement: S12 Fig — (A) Results of western blot analysis of proteins expressed in N. benthamiana leaves as shown in Fig 3B. (B) A full-size image of the western blot as shown in (A). (C)Histograms showing the number of GFP:OsHIPP19 and GFP:OsHIPP20 puncta structure in N. benthamiana cells in the presence of GUS, HA:AVR-Pii and AVR-PikD:HA. (TIFF) [file ppat.1012647.s012.tiff]

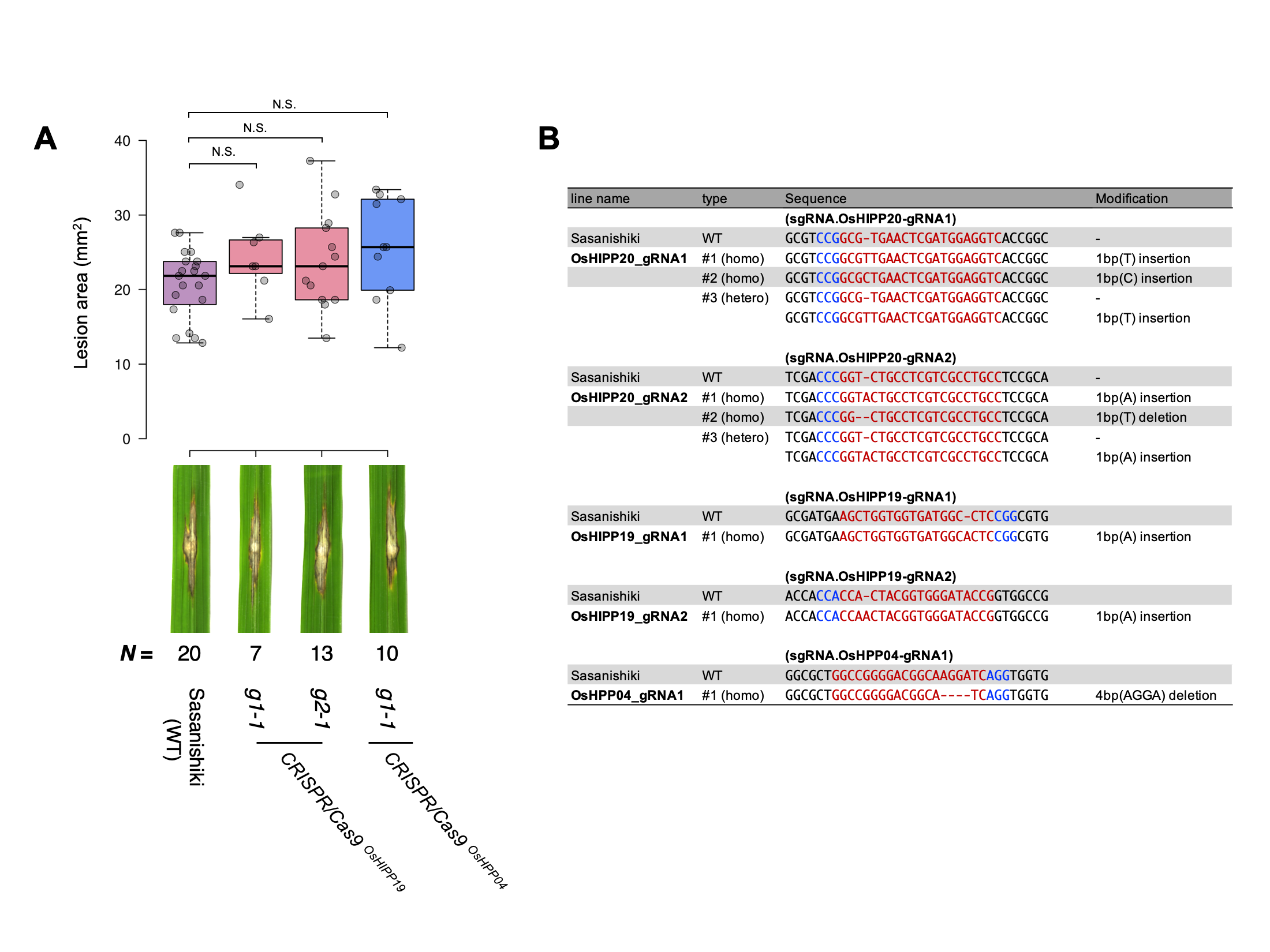

Supplement: S13 Fig — (A) A compatible M. oryzae isolate Sasa2 was punch inoculated onto the leaves of rice cultivar Sasanishiki as well as the sHMA-knockout lines of Sasanishiki (oshipp19#1, oshipp19#2 and oshpp04). Box plots show lesion area sizes in the rice lines (top). Statistical significance is shown after Wilcoxon rank sum test. Photos of typical lesions developed on the leaves after inoculation of M. oryzae (bottom). The number of leaves used for experiments are indicated below. (B) A table showing guide RNA and transgenic line nomenclature (left), location of guide RNA used for CRISPR/Cas9 mutagenesis (center) and the resulting nucleotide changes (center and right). PAM is indicated with blue and the sgRNA sequence is indicated with red letters. (TIFF) [file ppat.1012647.s013.tiff]

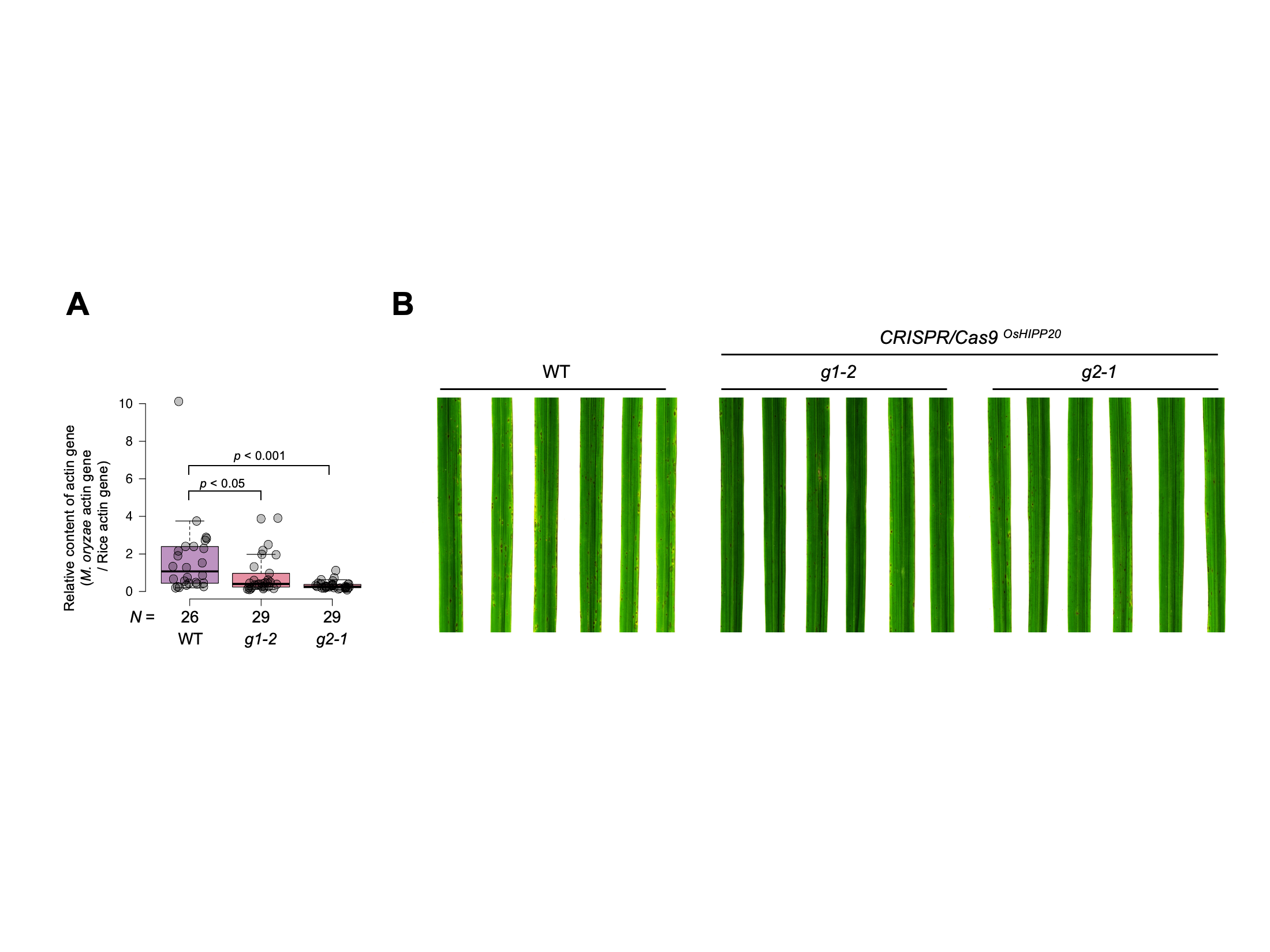

Supplement: S14 Fig — (A) Results of spray inoculation of M. oryzae Ken53-33 to the wild-type Sasanishiki (WT) and two OsHIPP20-knockout lines g1-2 and g2-1 (2nd replication). Statistical significance is shown after Wilcoxon rank sum test. (B) Images of leaves of Sasanishiki wild-type (WT) and OsHIPP20 knockout lines g1-2 and g2-1 4 days after spray inoculation of M. oryzae Ken53-33. (TIFF) [file ppat.1012647.s014.tiff]

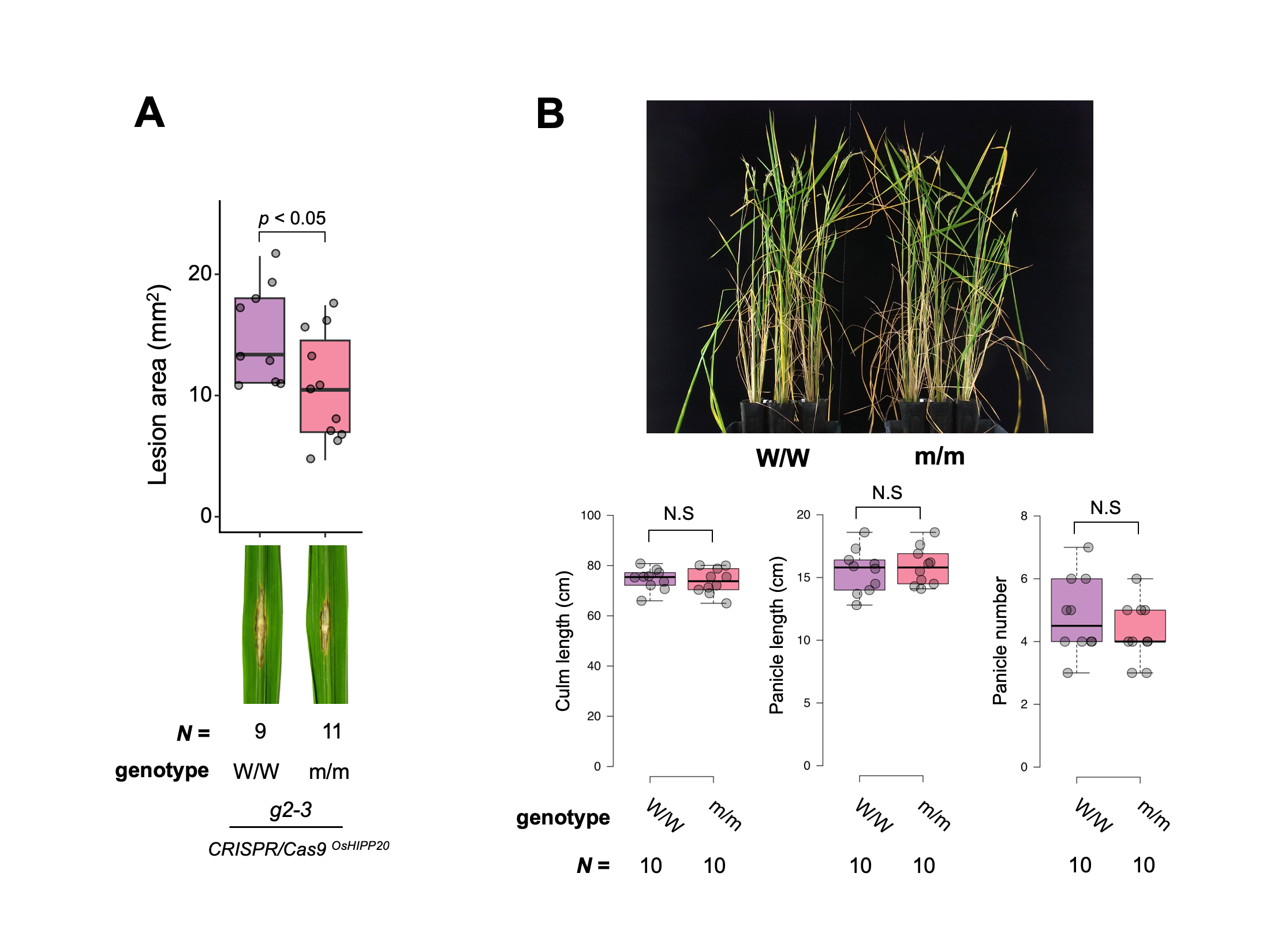

Supplement: S15 Fig — (A) Punch inoculation results of T2 generation of homozygous KO rice plants. Statistical significance is shown after Wilcoxon rank sum test. (B) No growth defect in OsHIPP20 + LOC_Os04g32290.1 KO line as compared to the wild type. Top: Overview of the W/W and m/m plants. Bottom: Box plot showing culm length (left), panicle length (center) and panicle number (right) of W/W and m/m plants. The numbers (N) below the graph show the number of individuals for which measurement was made. Statistical significance is shown after Wilcoxon rank sum test. (TIFF) [file ppat.1012647.s015.tiff]

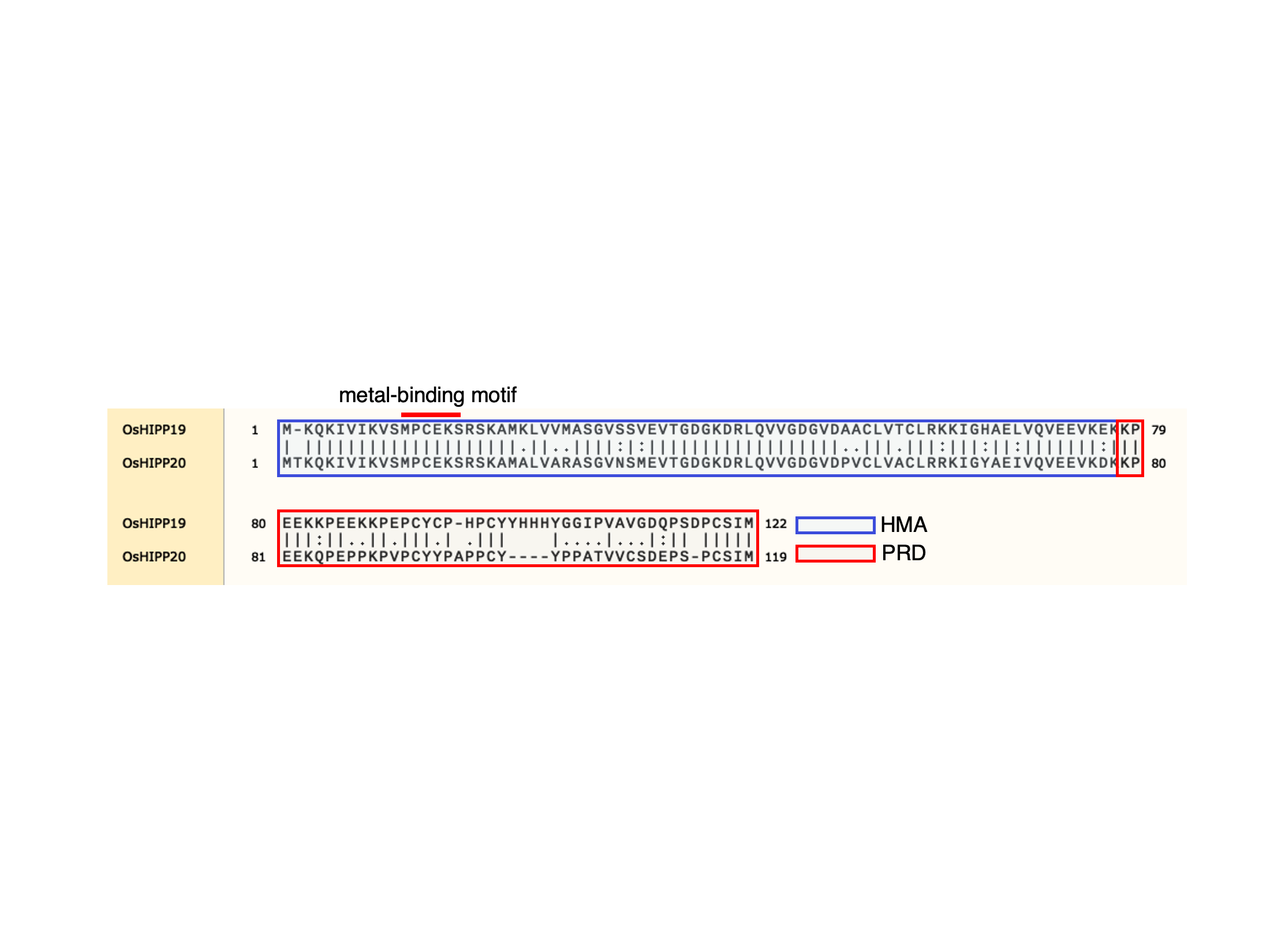

Supplement: S16 Fig — HMA domain and proline rich domain (PRD) are indicated by blue and red square, respectively. (TIFF) [file ppat.1012647.s016.tiff]
